# Multifunctional Phosphate Monomer Enabling LiNO_3_ Solvation and In Situ Formation of Flame‐Retardant Gel Polymer Electrolyte for High‐Voltage Lithium Metal Batteries

**DOI:** 10.1002/advs.202524165

**Published:** 2026-01-31

**Authors:** Lijun Ma, Lu Lu, Tianqi Xiang, Yaxin Wang, Jianjun Zhou, Lin Li

**Affiliations:** ^1^ College of Chemistry Beijing Normal University Beijing China; ^2^ College of Textiles & Clothing Qingdao University Qingdao China

**Keywords:** flame retardant, gel polymer electrolytes, in situ polymerization, LiNO_3_ solvation, lithium metal batteries

## Abstract

Preparing electrolytes that simultaneously enable high oxidation stability, interfacial compatibility, and intrinsic safety remains a major challenge for high‐voltage lithium metal batteries (LMBs). Herein, we report a phosphorus‐containing multifunctional monomer, ethyl di(2‐(methacryloyloxy)ethyl) phosphate, which enables both LiNO_3_ dissolution and in situ polymerization within a liquid electrolyte (LE) to form a flame‐retardant gel polymer electrolyte (GPE). The resulting GPE exhibits excellent ionic conductivity (3.19 × 10^−3^ S cm^−1^ at 25°C), a wide electrochemical stability window (> 4.6 V), and superior flame retardancy. The LiNO_3_ in GPE can promote the formation of Li_3_N and LiF in the solid electrolyte interphase (SEI) layer on the Li metal anode, facilitating Li^+^ transport and promoting dense and smooth Li deposition. When applied in Li||LiNi_0.6_Co_0.2_Mn_0.2_O_2_ and Li||LiNi_0.8_Co_0.1_Mn_0.1_O_2_ cells, the GPE system delivers remarkable cycling stability with capacity retentions of 83.1% after 400 cycles and 91.5% after 200 cycles, respectively. Spectroscopic and structural analyses reveal that the polymer matrix in GPE stabilizes cathode–electrolyte interfaces, mitigates transition‐metal dissolution, and suppresses Li/Ni cation mixing. This work establishes a molecular‐level electrolyte design strategy that integrates LiNO_3_ solvation, flame retardancy, and interfacial stabilization, offering a promising pathway toward safe, high‐voltage LMBs.

## Graphical abstract

A phosphorus‐containing multifunctional monomer is successfully synthesized to simultaneously enable the dissolution of lithium nitrate in ester‐based electrolytes and in situ form a flame‐retardant gel polymer electrolyte (GPE). The formed GPE effectively stabilizes the structures of both the cathode and anode, thereby achieving superior long‐term cycling performance and enhanced safety in high‐voltage lithium metal batteries.

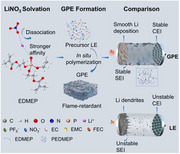

## 1 Introduction

With the rapid growth of portable electronics and electric vehicles, the demand for battery systems with high energy density and superior safety has become increasingly urgent [1, 2, 3]. Among various candidates, metallic lithium (Li) is regarded as the most promising anode material due to its exceptionally high theoretical capacity (3860 mAh g^−1^) and the lowest electrochemical potential (−3.04 V vs. the standard hydrogen electrode). These features make Li metal the only anode capable of achieving energy densities exceeding 500 Wh kg^−1^ [4, 5, 6, 7]. Despite these advantages, lithium metal batteries (LMBs) remain hindered by critical challenges, most notably the uncontrollable formation of Li dendrites and the associated safety risks. The high chemical reactivity of Li causes the formation of an unstable and inhomogeneous solid electrolyte interphase (SEI), leading to uneven Li^+^ flux and dendritic Li deposition during repeated plating/stripping processes. Consequently, dendritic Li growth results in continuous electrolyte consumption, rapid capacity decay, and shortened cycling life. More seriously, dendrites can penetrate the separator, causing internal short circuits. In addition, conventional organic liquid electrolytes (LEs) used in LMBs are volatile and highly flammable, posing severe fire and explosion hazards during thermal runaway or mechanical failure [8, 9, 10, 11, 12]. Therefore, dendrite free Li deposition and improving interfacial stability are essential prerequisites for the safe and practical application of high‐energy‐density LMBs.

Gel polymer electrolytes (GPEs), combining the merits of both LEs and solid electrolytes, have been one of the most promising candidates to address these challenges [13, 14, 15]. Additionally, the GPE with tailored chemical composition enables precise regulation of the composition and structure of the electrode/electrolyte interface [16, 17, 18]. Well‐designed GPEs can improve the safety and electrochemical stability of LMBs by reducing solvent evaporation and leakage, homogenizing ion flux, and mitigating dendritic Li deposition [19, 20, 21]. Furthermore, by copolymerizing flame‐retardant monomers, GPEs can be endowed with intrinsic fire‐resistant properties [7, 22]. Since the materials containing fluorine (F) and phosphorus (P) elements can effectively capture free radicals and quench flames, various F‐ and/or P‐containing monomers and polymers have been used to form flame‐retardant GPEs [23, 24]. Song et al. grafted fluoride‐containing moieties onto cyanoethyl poly(vinyl alcohol) chains and obtained a nonflammable cross‐linked GPE [25]. Jia et al. developed a copolymer containing hydroxyl, nitrile, and diethyl phosphonate groups, which can be in situ cross‐linked through a Ritter reaction, forming a nonflammable GPE [13]. Xu et al. introduced fluoroalkyl (‐CF_2_CF_2_‐) segments into phosphate cross‐linkers to design a fluorinated phosphate‐based GPE, where the incorporation of fluoroalkyl and phosphate groups synergistically enhanced the flame retardancy [7].

Although GPEs can homogenize Li^+^ flux and partially suppress dendritic growth, achieving completely dendrite‐free Li deposition remains difficult, particularly during the initial cycling stages. To further stabilize the Li metal anode, introducing functional additives into the electrolyte has proven to be an effective strategy [26, 27, 28, 29]. Among various additives, lithium nitrate (LiNO_3_) has demonstrated excellent ability to suppress dendrite formation in ether‐based electrolytes. However, its limited solubility in ester‐based electrolytes restricts its application in high‐energy‐density LMBs [30, 31, 32]. Considerable efforts have been made to enhance LiNO_3_ solubility, such as using high‐dielectric‐constant solvents [26, 33, 34], Lewis acid additives [35, 36, 37, 38], or multivalent linear esters [39, 40, 41]. Among these approaches, the use of unsaturated multivalent linear esters is particularly promising, as they can not only facilitate LiNO_3_ incorporation but also undergo in situ cross‐linking to form LiNO_3_‐containing GPEs. Moreover, when these esters contain phosphorus elements, the resulting GPEs exhibit flame‐retardant characteristics. Therefore, developing phosphorus‐containing, LiNO_3_‐loaded GPEs is expected to achieve both dendrite suppression and flame retardancy, improving the safety performance of LMBs.

Herein, a phosphorus‐containing multivalent acrylate was synthesized via a nucleophilic substitution reaction and employed as an electrolyte additive. This compound serves both as a co‐solvent for dissolving LiNO_3_ in ester‐based electrolytes and as a cross‐linkable monomer for in situ formation of a flame‐retardant GPE. The resulting LiNO_3_‐loaded GPE effectively modulates the Li^+^ solvation structure, facilitating the formation of a uniform and robust SEI enriched with Li_3_N and LiF. Such a tailored SEI suppresses dendritic Li growth, mitigates electrolyte decomposition, and enhances interfacial stability. In addition, the LiNO_3_‐containing GPE contributes to the structural stabilization of high‐voltage ternary cathodes (e.g., LiNi_0.6_Co_0.2_Mn_0.2_O_2_ (NCM622)) by suppressing transition‐metal dissolution and interfacial degradation. This integrated strategy, combining flame retardancy, in situ GPE formation, and interfacial regulation together, can synergistically improve the cycling stability of both Li||Li symmetric cells and Li||NCM622 batteries. Overall, this work provides a promising route toward high‐safety, long‐life LMBs.

## 2 Results and Discussion

### 2.1 Synthesizing and Characterizing Monomer

**FIGURE 1:**
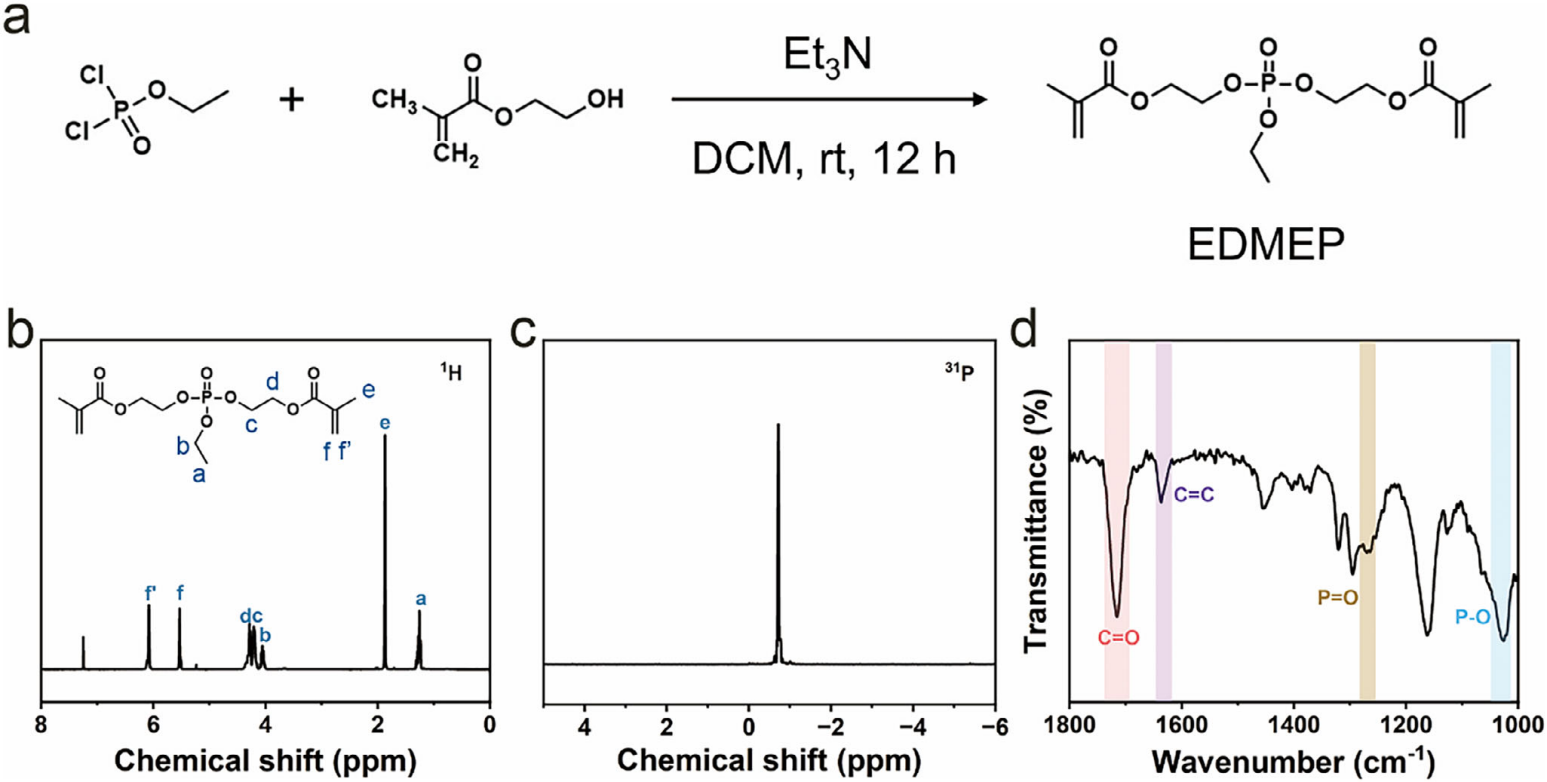
Synthesizing and characterizing EDMEP. (a) Synthesizing route for EDMEP. (b) ^1^H and (c) ^31^P NMR spectra of EDMEP. (d) FTIR profile of EDMEP.

A multifunctional phosphate monomer, ethyl di(2‐(methacryloyloxy)ethyl) phosphate (EDMEP), was synthesized via a nucleophilic substitution reaction between ethyl dichlorophosphate and 2‐hydroxyethyl methacrylate, as illustrated in Figure 1a. The successful synthesis of EDMEP was verified by ^1^H and ^31^P nuclear magnetic resonance (NMR) spectroscopy (Figure 1b,c). Detailed chemical shifts are provided in the sample preparation section of Supporting Information. In the ^1^H NMR spectrum, the integration ratio of the peak (***e***) at 1.86 ppm to that (***a***) at 1.25 ppm is approximately 2:1, confirming that chlorine atoms in ethyl dichlorophosphate were substituted by methacryloyloxyethyl groups. These NMR observations are further supported by Fourier transform infrared (FTIR) spectroscopy (Figure 1d). The absorption bands at 1716 and 1635 cm^−1^ correspond to the stretching vibrations of C═O and C═C bonds, respectively. In addition, characteristic peaks at 1263 and 1024 cm^−1^ are assigned to P═O and P─O stretching vibrations of the phosphate moiety [42]. Collectively, the NMR and FTIR analyses unambiguously confirm the successful synthesis and structural integrity of EDMEP.

### 2.2 Co‐Solvent for LiNO_3_

It is well established that LiNO_3_ is hardly soluble in ester‐based LEs [35, 40]. As shown in Figure S1a, when 1.0 wt.% LiNO_3_ is added to the ester electrolyte, it remains undissolved regardless of the mixing method, and a visible precipitate accumulates at the bottom of the vial. In contrast, upon introducing 5.0 wt.% EDMEP, the LiNO_3_ completely dissolves after stirring at 60°C for 1 h (Figure S1b), demonstrating that EDMEP serves effectively as a co‐solvent to enhance LiNO_3_ solubility.

**FIGURE 2:**
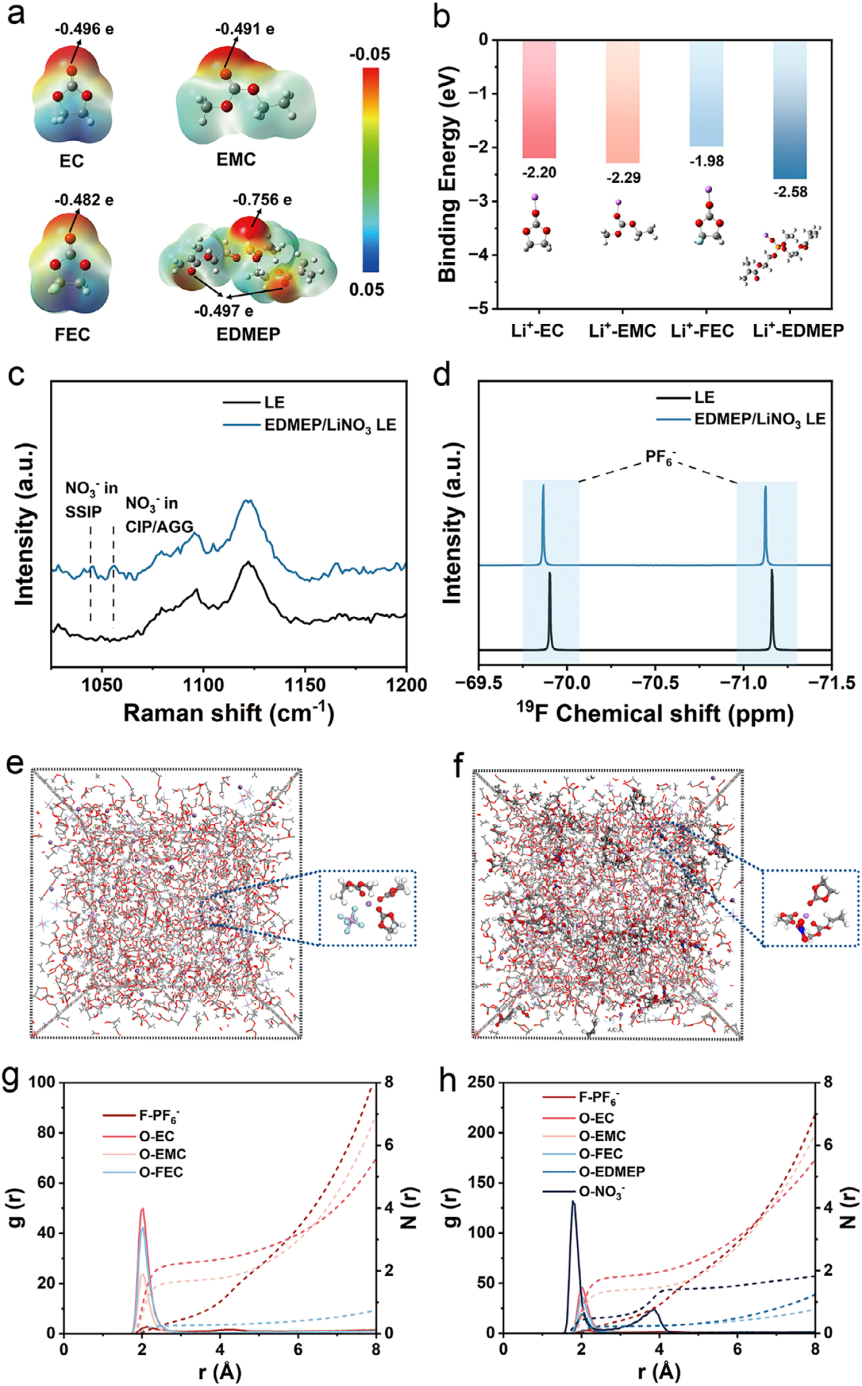
(a) Simulated ESP mapping of EDMEP and solvent molecules in the electrolyte and (b) the binding energy with Li^+^. (c) Raman spectra of the LE and EDMEP/LiNO_3_‐containing LE. (d) ^19^F NMR spectra of the LE and EDMEP/LiNO_3_‐containing LE. Snapshots of the MD simulation of the (e) LE and (f) EDMEP/LiNO_3_‐containing LE (The magnified sections depict the first Li^+^ solvation shell structures). The radial distribution function (*g*(r), solid line) and coordination number (*N*(r), dashed line) for (g) LE and (h) EDMEP/LiNO_3_‐containing LE.

To gain further insight into the LiNO_3_ solvation mechanism, density functional theory (DFT) calculations were performed. The electrostatic potential (ESP) mapping (Figure 2a) reveals that the carbonyl oxygen atom in the phosphonate group of EDMEP exhibits a more negative electrostatic potential (−0.756 e) compared with those in ester‐based solvent molecules, indicating a stronger affinity toward Li^+^. Correspondingly, the calculated binding energy between Li^+^ and EDMEP is significantly higher than that between Li^+^ and conventional carbonate solvents (Figure 2b), suggesting that EDMEP can effectively weaken the Li^+^‐NO_3_^−^ coordination, thereby facilitating LiNO_3_ dissolution. In light of previous work by Wang et al. [40], who reported enhanced LiNO_3_ solubility in saturated multivalent linear esters through a low‐entropy‐penalty mechanism, it is likely that the multivalent ester chains in EDMEP also show the entropy penalty during solvation. This synergistic effect of strong Li^+^ coordination and favorable entropy collectively accounts for the markedly improved LiNO_3_ solubility in the presence of EDMEP.

To further elucidate the influence of EDMEP and LiNO_3_ on the Li^+^ solvation structure, Raman spectroscopy was conducted to compare the spectral profiles of the pristine LE and the EDMEP/LiNO_3_‐containing LE (Figure 2c). Compared with the spectrum of the pristine LE, two additional characteristic bands appear at 1044 and 1055 cm^−1^ in the EDMEP/LiNO_3_ system. The band at 1044 cm^−1^ corresponds to NO_3_^−^ species in the form of solvent‐separated ion pairs (SSIP), while the band at 1055 cm^−1^ is attributed to contact ion pairs (CIP) or anion aggregates (AGG) [39]. The emergence of these peaks indicates the incorporation of NO_3_^−^ into the Li^+^ solvation sheath, forming CIP/AGG‐type complexes. Such coordinated structures facilitate the synchronous migration of Li^+^ and NO_3_^−^. Further evidence for the altered solvation environment is provided by ^19^F NMR spectroscopy (Figure 2d). In the EDMEP/NO_3_^−^‐containing LE, the resonance signal of the PF_6_^−^ anion shifts downfield, suggesting a deshielding effect induced by the presence of LiNO_3_. This observation implies that NO_3_^−^ partially replaces PF_6_^−^ in the primary Li^+^ solvation shell, thereby weakening the Li^+^‐PF_6_^−^ coordination [43]. Collectively, the Raman and ^19^F NMR analyses demonstrate that EDMEP effectively modulates the Li^+^ solvation environment, enabling NO_3_^−^ participation in the solvation sheath.

To gain molecular‐level insights into the Li^+^ solvation environment, molecular dynamics (MD) simulations were performed for both the pristine LE and the EDMEP/LiNO_3_‐containing LE. Representative snapshots and the corresponding first‐shell Li^+^ solvation structures are presented in Figure 2e,f. Compared with the pristine LE, the EDMEP/LiNO_3_ system clearly shows the participation of NO_3_^−^ in the first Li^+^ solvation shell, confirming the structural modification induced by EDMEP. As shown in Figure 2g, in the pristine LE, the main Li^+^─O coordination peak appears at 2.02 Å, characteristic of typical ester‐type solvation. Quantitative coordination analysis reveals that ethylene carbonate (EC) dominates the inner solvation shell (coordination number (CN) = 2.30), followed by ethyl methyl carbonate (EMC) (CN = 1.70) and PF_6_^−^ (CN = 0.60). Upon the introduction of EDMEP/LiNO_3_, a new sharp coordination peak emerges at 1.77 Å, corresponding to Li^+^─NO_3_^−^ interactions, while the Li^+^─O coordination distance (∼2.02 Å) remains unchanged (Figure 2h). The appearance of this shorter Li^+^─NO_3_^−^ distance signifies that NO_3_^−^ occupies the innermost layer of the solvation sheath. Meanwhile, the CN value of PF_6_^−^ decreases from 0.60 to 0.38, indicating a competitive replacement of PF_6_^−^ by NO_3_^−^ in the Li^+^ solvation structure. Structural reorganization induced by EDMEP also weakens solvent coordination: the CN values of EC and EMC decrease from 2.30 to 1.80 and 1.70 to 1.37, respectively. Furthermore, the calculated binding energy of the first Li^+^ solvation shell becomes more negative in the EDMEP/LiNO_3_‐containing system than in the pristine LE (Figure S2), suggesting a thermodynamically more stable solvation configuration and more favorable Li^+^ transport kinetics [44]. Collectively, the MD simulations demonstrate that EDMEP facilitates the incorporation of NO_3_^−^ into the primary Li^+^ solvation shell, forming a NO_3_^−^‐dominated coordination environment. This configuration enables the synchronous migration of Li^+^ and NO_3_^−^.

### 2.3 Constructing GPE Containing LiNO_3_

**FIGURE 3:**
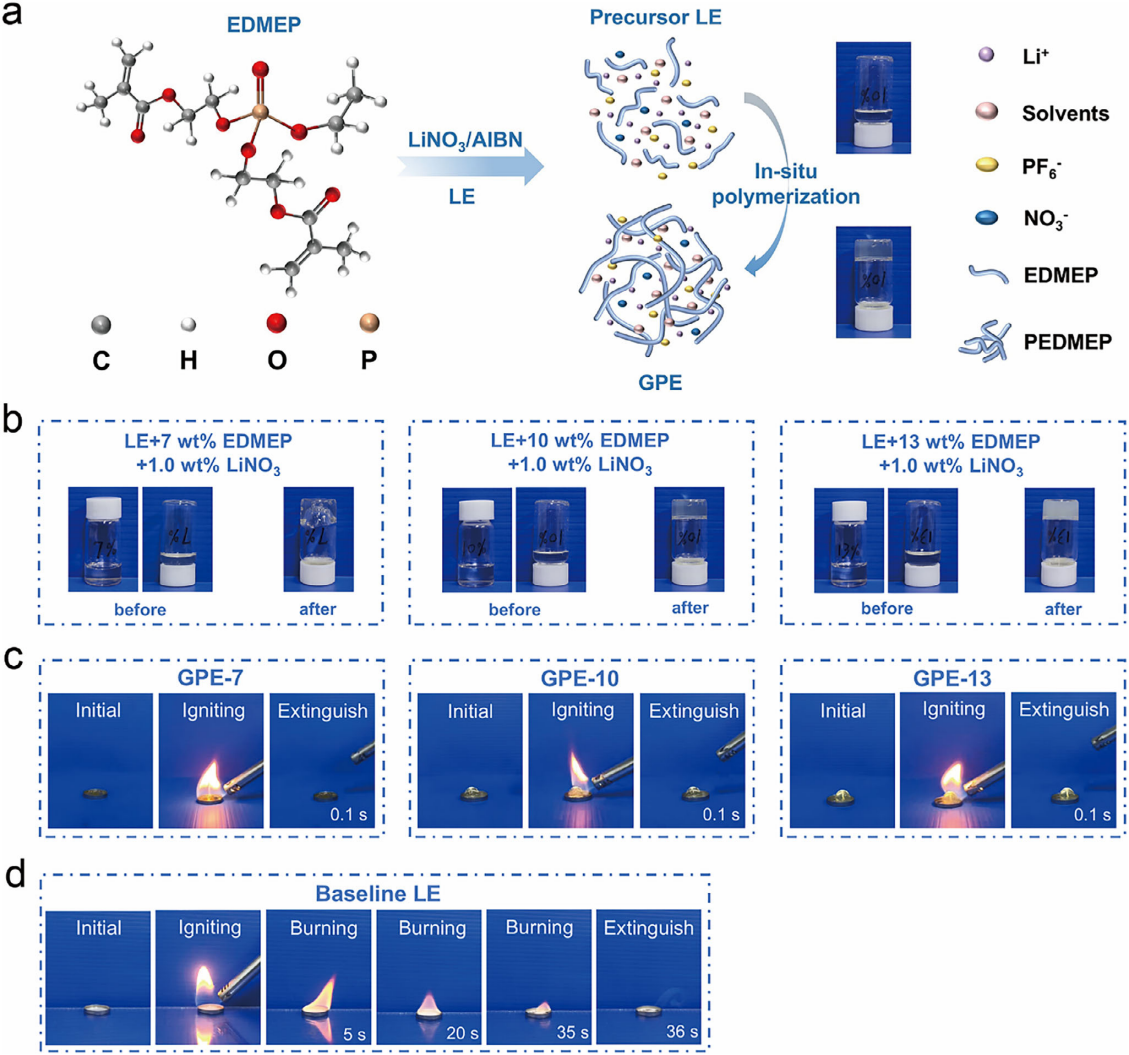
(a) Schematic illustration of in situ polymerization of GPE. (b) Optical images of precursor LEs and GPEs. The combustion experiments of (c) GPE‐7, GPE‐10, GPE‐13, and (d) pristine LE.

EDMEP contains two acrylate functional groups, enabling its use as a cross‐linkable monomer to fabricate LiNO_3_‐containing GPEs via free‐radical polymerization (Figure 3a; Figure S3). Precursor LEs were prepared by dissolving EDMEP (7.0, 10.0, or 13.0 wt.%), LiNO_3_ (1.0 wt%), and the initiator azobisisobutyronitrile (AIBN, 0.1 wt.%). After complete dissolution of LiNO_3_, the precursor LEs appear optically transparent, as shown in Figure 3b. Upon heating at 65°C for 8 h, these transparent precursor LEs undergo in situ polymerization, transforming into GPEs. When the EDMEP concentration is 7.0 wt.%, the resulting product (denoted as GPE‐7) remains slight fluidity, suggesting partial gelation. As the EDMEP content increases to 10.0 and 13.0 wt.%, the systems lose fluidity and convert into self‐standing, non‐flowable gels (denoted as GPE‐10 and GPE‐13, respectively) (Figure 3b). Since GPE‐10 exhibits sufficient mechanical integrity without compromising processability, it was selected for subsequent investigations. The FTIR spectra provide direct evidence of successful polymerization (Figure S4). The characteristic band at 1635 cm^−1^, corresponding to the C═C stretching vibration of EDMEP, completely disappears in GPE‐10, confirming that the double bonds were consumed during polymer network formation [13]. Thermogravimetric analysis (TGA) further confirms the enhanced thermal stability of GPE‐10 and the cross‐linked polymer matrix (PEDMEP) (Figure S5). Compared with LE, GPE‐10 exhibits almost no weight loss at the gelation temperature of 65°C, indicating that the volatilization of solvent molecules is effectively retarded, which suggests that the solvent molecules are well confined within the polymer framework. Meanwhile, PEDMEP shows negligible weight loss even at a temperature as high as 200°C, demonstrating excellent thermal stability. These results verify that EDMEP undergoes efficient radical polymerization to form a stable cross‐linked GPE framework capable of immobilizing the liquid exponents with enhanced thermal stability.

### 2.4 Flame Retardant Property of GPE

Phosphorus‐containing compounds are well known for their intrinsic flame‐retardant properties [45, 46]. Notably, the GPEs formed via the polymerization of EDMEP within LEs exhibit remarkably enhanced flame resistance compared to their liquid precursors. Combustion tests reveal that GPE‐7, GPE‐10, and GPE‐13 display outstanding self‐extinguishing behavior (Figure 3c; Videos S1−S3). Upon removal of the ignition source, the flames were quenched almost instantaneously. In contrast, the pristine LE and precursor LE containing 10.0 wt.% EDMEP exhibit significantly longer burning times of 36 and 23 s, respectively (Figure 3d; Videos S4 and S5 and Figure S6a). However, to achieve a comparable level of self‐extinguishing performance as that of GPEs, the EDMEP concentration in the LE must be increased to 50.0 wt.% (Figure S6b and Video S6), indicating that the flame‐retardant efficiency of EDMEP is greatly enhanced in the cross‐linked polymer framework. This improvement can be attributed to two synergistic effects. First, the 3D polymer network effectively confines the liquid electrolyte, thereby restricting the volatilization and diffusion of combustible components under high‐temperature conditions [47]. Second, during combustion, phosphorus‐containing fragments in the polymer backbone can generate PO· and HPO· radicals, which capture high‐energy radicals such as H· and OH· in the flame zone, thus interrupting the chain reaction of combustion [7]. Collectively, these results demonstrate that the EDMEP‐derived GPEs exhibit excellent flame retardancy and rapid self‐extinguishing capability, providing a promising safety enhancement strategy for next‐generation high‐energy‐density LMBs.

### 2.5 Electrochemical Properties

**FIGURE 4:**
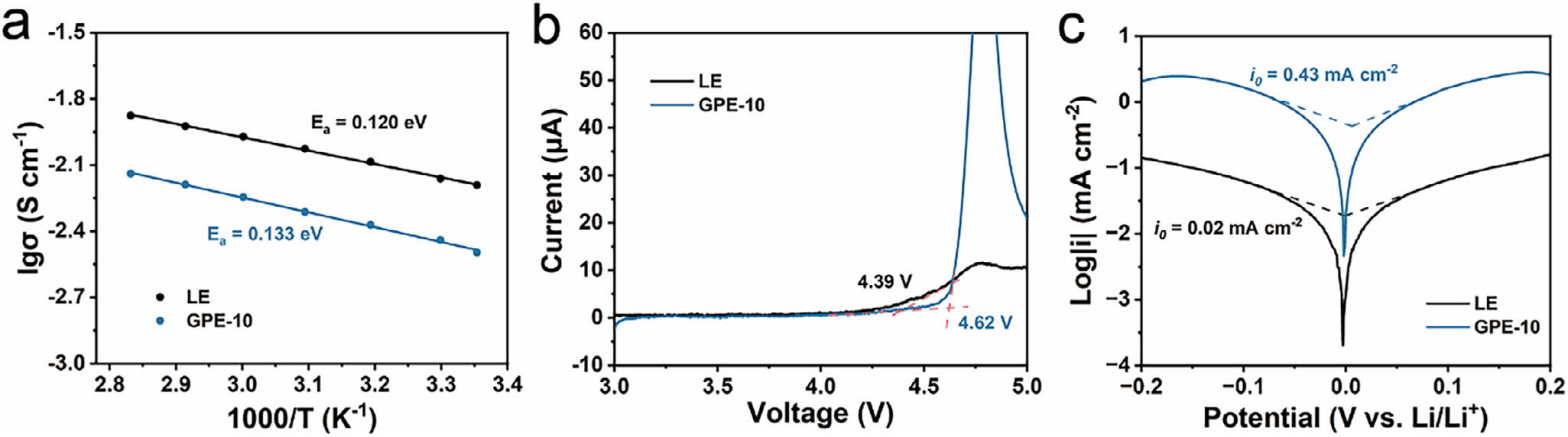
(a) Ionic conductivity and (b) Linear sweep voltammetry curves of LE and GPE‐10. (c) Tafel plots for Li plating/stripping in LE and GPE‐10.

The ionic conductivity of the electrolytes was evaluated by electrochemical impedance spectroscopy (EIS) using stainless steel (SS)||SS cells (Figure S7). At 25°C, the conductivities of LE and GPE‐10 are 6.44 and 3.19 mS cm^−1^, respectively. With increasing temperature, both systems exhibit enhanced ion mobility, following the Arrhenius relationship (Figure 4a). The calculated activation energies (*E*_a_) for LE and GPE‐10 are 0.120 and 0.133 eV, respectively. The slightly higher *E*_a_ for GPE‐10 suggests a modestly greater energy barrier for Li^+^ migration within the cross‐linked polymer matrix, likely due to partial restriction of solvent motion by the network structure. Nevertheless, GPE‐10 still maintains high ionic conductivity suitable for practical LMBs. Beyond ionic mobility, the Li^+^ transference number ($t_{Li^{+}}$) was determined via combined EIS and chronoamperometric measurements (Figure S8). The $t_{Li^{+}}$ value for GPE‐10 reaches 0.61, nearly twice that of LE (0.33). This enhancement originates from specific interactions between the PEDMEP polymer chains and the anions, which effectively immobilize PF_6_^−^ and facilitate preferential Li^+^ migration. A higher $t_{Li^{+}}$ mitigates concentration polarization and reduces LiPF_6_ decomposition at the anode interface, thereby promoting a more stable SEI and improving the long‐term cycling stability of LMBs [13].

The electrochemical stability window was investigated using linear sweep voltammetry (LSV). As shown in Figure 4b, GPE‐10 exhibits a significantly higher oxidative decomposition potential (4.62 V) compared to the LE (4.39 V), indicating its ability to sustain operation under high‐voltage conditions. This improvement arises from the polymer matrix's strong solvent‐binding ability, which suppresses solvent oxidation at elevated potentials [48]. The cyclic voltammetry (CV) profile of GPE‐10 (Figure S9) displays a distinct reduction peak at ∼1.0 V during the first cycle, corresponding to the reduction of NO_3_^−^. The absence of this peak in subsequent scans confirms that NO_3_^−^ participates in forming a stable, inorganic‐rich SEI layer, primarily composed of Li_3_N [43].

Furthermore, the exchange current density (*i*_0_) of the Li|GPE‐10|Li symmetric cell is 0.43 mA cm^−2^, markedly higher than that of the LE cell (0.02 mA cm^−2^) (Figure 4c), indicating significantly improved charge‐transfer kinetics. This enhancement can be attributed to the highly conductive SEI generated by the reduction of NO_3_^−^, which facilitates Li^+^ transport across the interface and promotes uniform Li deposition. Overall, the integration of EDMEP and LiNO_3_ into GPE‐10 not only preserves high ionic conductivity and increases Li^+^ transference efficiency but also broadens the electrochemical stability window, demonstrating a synergistic improvement in ion transport, interfacial stability, and electrochemical reaction kinetics.

### 2.6 Application in Li||Li Symmetric Cells

**FIGURE 5:**
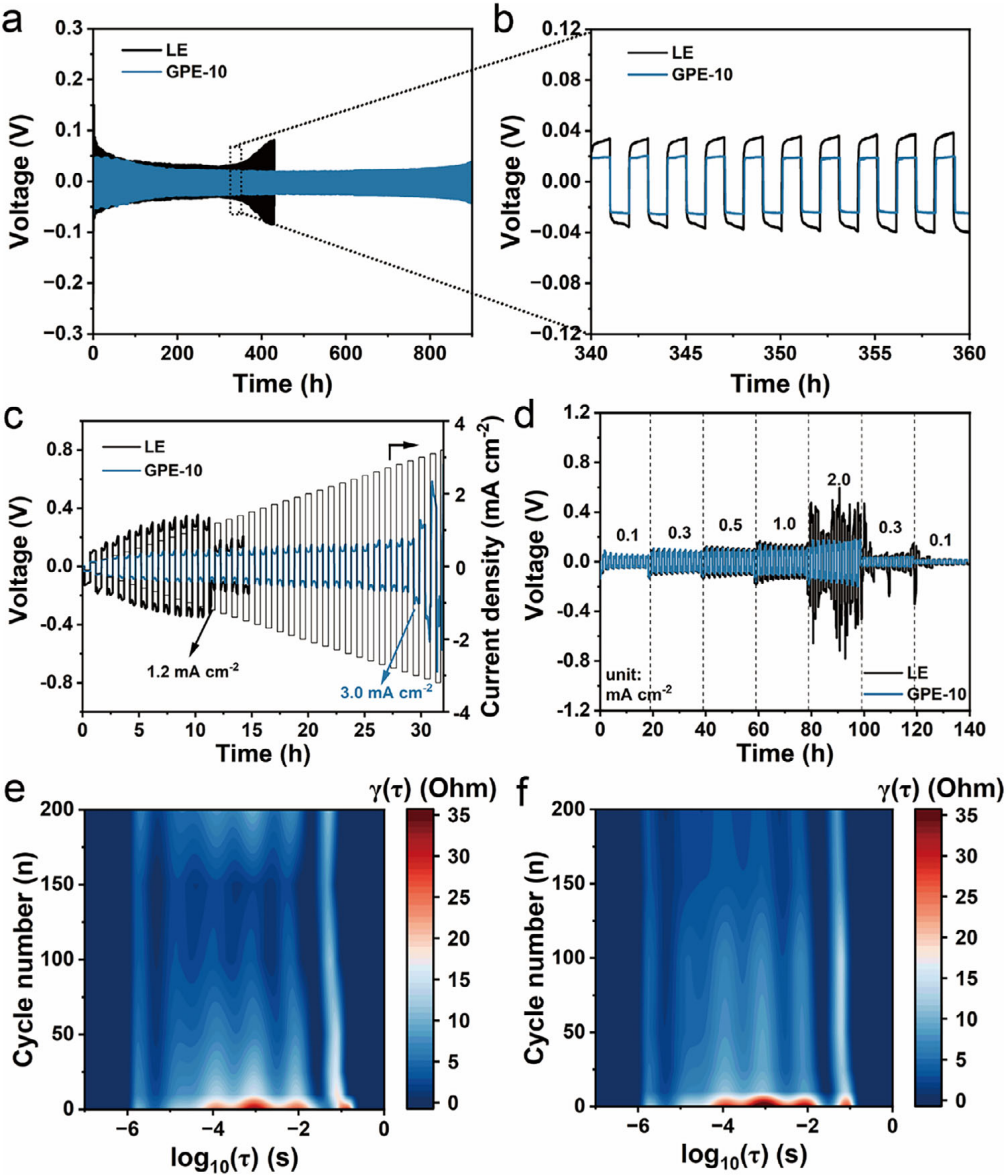
(a) Voltage profiles of Li||Li symmetric cells at 0.2 mA cm^−2^ and 0.2 mAh cm^−2^. (b) Enlarged voltage profiles. (c) The critical current densities and (d) rate performances of Li||Li symmetric cells. Contour maps of DRT spectra of Li||Li cells with (e) LE and (f) GPE‐10 cycling at 0.2 mA cm^−2^ and 0.2 mAh cm^−2^.

The Li plating/stripping behavior of the electrolytes was investigated using Li||Li symmetric cells. As illustrated in Figure 5a, the Li|LE|Li cell exhibits pronounced overpotential hysteresis after approximately 320 h, signifying increasing interfacial instability during prolonged cycling. In contrast, the Li|GPE‐10|Li cell maintains a remarkably stable overpotential for over 800 h, reflecting superior interfacial robustness. The magnified voltage profiles (Figure 5b) display nearly square‐shaped waves with a small polarization of ∼20 mV for GPE‐10, indicative of uniform Li plating/stripping. Conversely, the LE cell shows significant voltage fluctuation (>50 mV) and irregular waveforms, suggesting continuous SEI rupture and reconstruction during cycling. The superior interfacial stability of GPE‐10 is further validated at elevated current densities and areal capacities (Figure S10). The critical current density (CCD) for stable Li deposition in the GPE‐10 system reaches 3.0 mA cm^−2^, substantially higher than that of LE (1.2 mA cm^−2^), confirming that the gel electrolyte effectively suppresses dendritic Li growth (Figure 5c). Rate‐performance tests (Figure 5d) further reveal that the GPE‐10‐based symmetric cell sustains lower overpotentials than the LE cell once the current density exceeds 0.5 mA cm^−2^. Even at 2.0 mA cm^−2^, GPE‐10 maintains stable voltage profiles, whereas LE exhibits severe polarization hysteresis, indicative of uneven Li plating and unstable interfacial dynamics. These results collectively demonstrate that GPE‐10 significantly enhances Li^+^ transport uniformity, mitigates polarization, and stabilizes the Li/electrolyte interface.

To gain further insights, the interfacial resistance evolution was monitored by EIS and analyzed using the distribution of relaxation times (DRT) technique (Figure 5e,f; Figure S11) [49]. Initially, both systems show a gradual decrease in resistance with cycling, suggesting the formation of SEI layer. However, after 150 cycles, the LE cell exhibits a sharp increase in resistances across all relaxation times, reflecting continuous degradation of the SEI layer and electrolyte depletion. In contrast, the GPE‐10 cell retains low and stable impedance throughout prolonged cycling, confirming superior interfacial and charge‐transfer stability. Equivalent‐circuit fitting (Figure S11c,d) further supports this trend. Although the GPE‐10 cell initially exhibits slightly higher *R*_SEI_ and *R*_ct_ values due to its higher viscosity and lower ionic conductivity, both parameters remain small and stable even after 150 cycles. Whereas *R*_SEI_ and *R*_ct_ values increase dramatically in the LE system, which may originate from the unstable SEI and severe parasite reactions [43]. This contrast indicates that GPE‐10 effectively suppresses parasitic reactions and preserves interfacial integrity over long‐term operation. The surface morphology of cycled Li electrodes (Figure S12) provides direct visual confirmation. The Li electrode retrieved from the GPE‐10 cell exhibits a dense, smooth, and uniform Li deposition layer, free of voids or dendritic protrusions. Conversely, the LE cell shows porous and dendritic Li deposits with extensive cracking, which significantly increases the effective surface area and accelerates electrolyte decomposition. This morphological evidence is consistent with the electrochemical observations of stable overpotential and low impedance evolution, conclusively verifying that the GPE‐10 effectively suppresses dendrite growth, mitigates parasitic reactions, and stabilizes the Li metal interface during repeated cycling.

**FIGURE 6:**
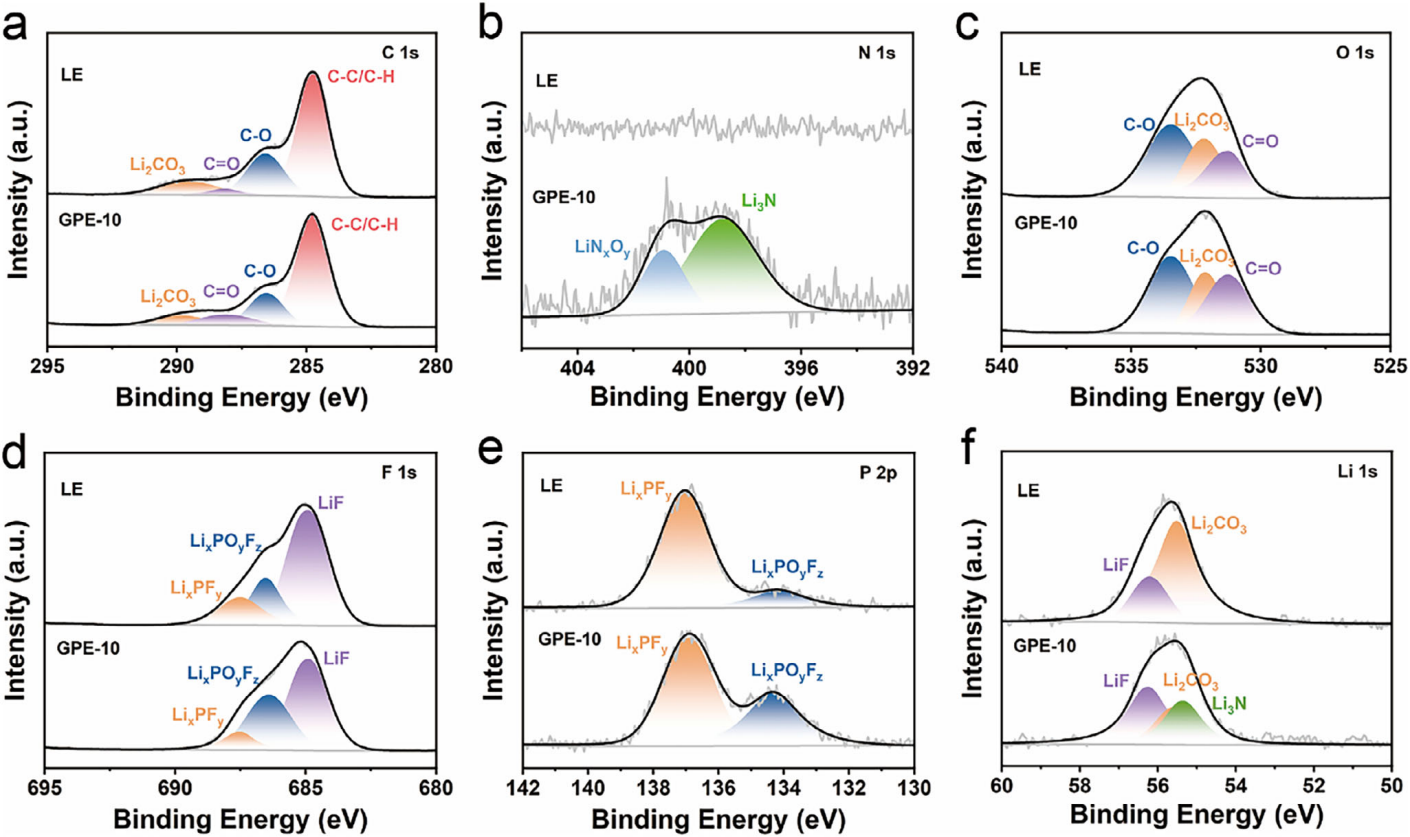
XPS spectra of (a) C 1s, (b) N 1s, (c) O 1s, (d) F 1s, (e) P 2p, and (f) Li 1s of Li anode surface from the Li||Li symmetric cells with LE and GPE‐10 after 100 cycles.

X‐ray photoelectron spectroscopy (XPS) was conducted to characterize the valence state of the elements on the Li metal surfaces of the Li||Li symmetric cells. The survey spectra (Figure S13) were calibrated using the C 1s peak at 284.8 eV. Distinct signals corresponding to Li_2_CO_3_ (C 1s: 289.8 eV; O 1s: 532.0 eV; Li 1s: 55.5 eV) are observed in both systems, representing the organic carbonate species typically generated from solvent decomposition [13]. Notably, the ratio of Li_2_CO_3_ on the Li anode cycled in the Li|GPE‐10|Li cell (8.2%) is significantly lower than that in the Li|LE|Li cell (11.9%) (Figure 6a), implying that the polymer matrix in GPE‐10 effectively suppresses solvent reduction and mitigates organic component accumulation in the SEI. New peaks centered at 398.9 eV (N 1s) and 55.4 eV (Li 1s) are detected exclusively on the Li surface from the GPE‐10 cell, which are assigned to Li_3_N species. The presence of Li_3_N confirms the nitrate reduction, consistent with the CV results, and indicates the formation of a Li_3_N‐enriched SEI layer. Due to its high Li^+^ ionic conductivity, Li_3_N facilitates rapid Li^+^ transport and uniform Li deposition, thereby reducing interfacial polarization [13, 50]. Additionally, prominent peaks at 56.3 eV (Li 1s) and 684.9 eV (F 1s) correspond to LiF, while peaks at 686.5 eV (F 1s) and 134.3 eV (P 2p) are assigned to Li_x_PO_y_F_z_ and peaks at 687.5 eV (F 1s) and 137.0 eV (P 2p) are attributed to Li_x_PF_y_, respectively. As illustrated in Figure 6d−f, the Li anode cycled in GPE‐10 exhibits a much higher ratio of LiF and Li_x_PO_y_F_z_ relative to Li_x_PF_y_, opposite to the trend observed in the LE system. Since Li_x_PF_y_ is the primary reduction product of LiPF_6_ [13], its suppression in the GPE‐10 cell suggests that the polymeric matrix and nitrate additive jointly inhibit LiPF_6_ decomposition. The enriched LiF and Li_x_PO_y_F_z_ phases are particularly beneficial: LiF forms an electron‐insulating yet ion‐permeable barrier, preventing further electrolyte breakdown, while Li_x_PO_y_F_z_ contributes high Li^+^ conductivity and mechanical robustness to the interphase [13, 51]. Collectively, the XPS results demonstrate that the LiNO_3_‐containing GPE effectively tailors the SEI composition toward an inorganic‐rich architecture composed predominantly of Li_3_N‐LiF‐Li_x_PO_y_F_z_ species. Such an ionically conductive interphase ensures homogeneous Li^+^ flux distribution, promotes dense and smooth Li deposition, and significantly enhances the interfacial stability of the Li metal anode, thereby underpinning the superior electrochemical performance of the GPE‐based system.

To further elucidate the potential reduction of electrolyte components on the Li metal anode, DFT calculations were performed. The results indicate that the lowest unoccupied molecular orbital (LUMO) energy levels of both PEDMEP and LiNO_3_ are considerably lower than those of the solvent molecules (Figure S14), suggesting their preferential reduction and involvement in the SEI formation. The reduction of PEDMEP facilitates the formation of a polymer‐derived SEI characterized by an infinite molecular weight polymer matrix, which enhances the stability of the SEI layer. Concurrently, the reduction of LiNO_3_ leads to the generation of a Li_3_N‐rich SEI. Given that NO_3_^−^ anions reside in close proximity to Li^+^ cations within the primary solvation shell (Figure 2h), they migrate along with Li^+^ and undergo reduction prior to LiPF_6_, thereby suppressing further decomposition of LiPF_6_. The resulting hybrid SEI, enriched with polymer matrix and Li_3_N, contributes to a more robust interface, effectively stabilizing the Li metal surface and curtailing continuous decomposition of liquid electrolyte components—a conclusion consistent with XPS analysis.

### 2.7 Application in Li||NCM Batteries

**FIGURE 7:**
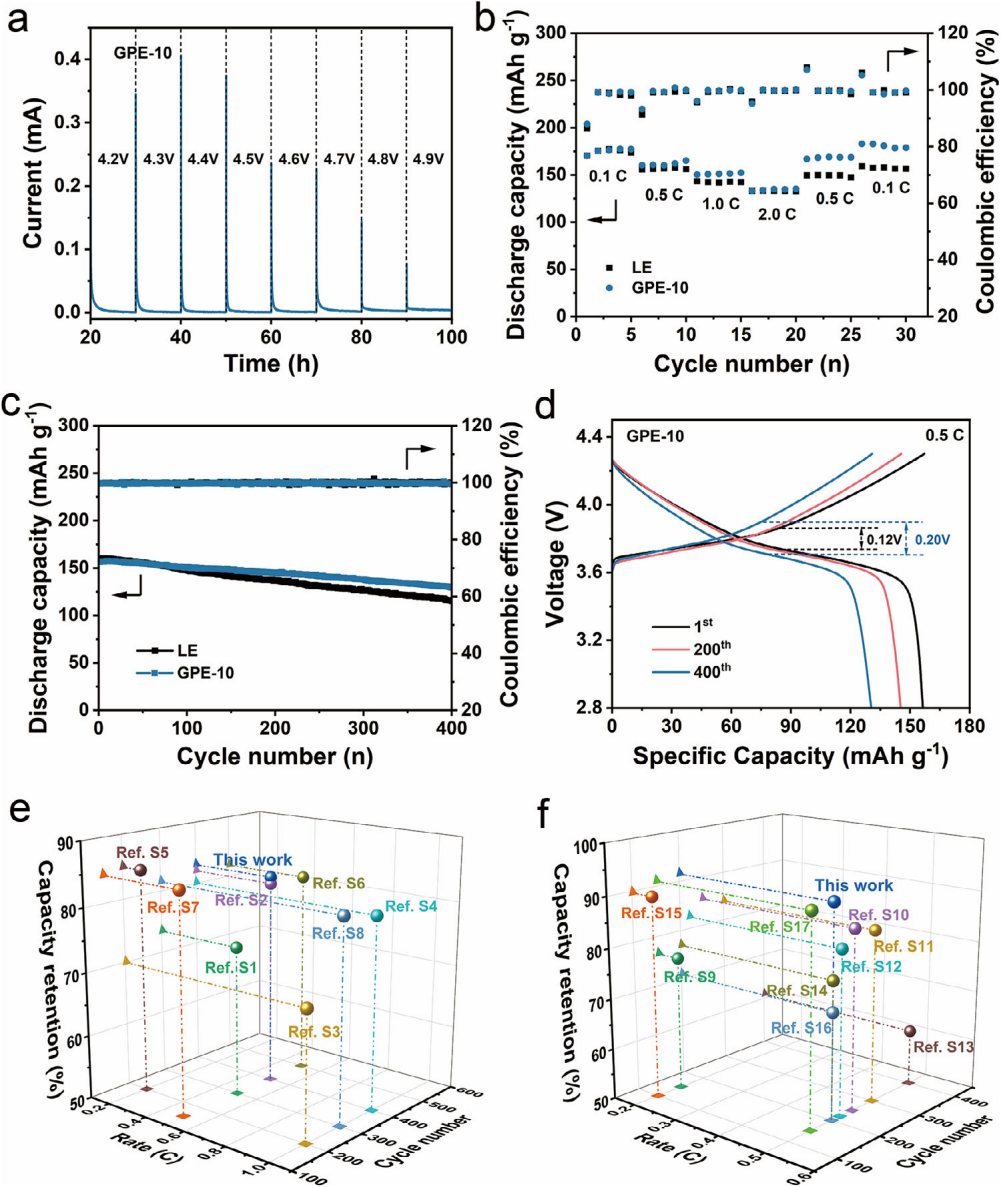
(a) Electrochemical floating test of GPE‐10. (b) *C*‐rate and (c) long‐term cycling performances of Li||NCM622 batteries. (d) Charge–discharge profiles of Li|GPE‐10|NCM622 battery. Comparative 3D images of the electrochemical performances of (e) Li||NCM622 and (f) Li||NCM811 batteries assembled with GPE‐10 and other previously reported polymer electrolytes.

To evaluate the compatibility of the polymer electrolyte with high‐voltage cathodes, Li||NCM622 batteries were assembled using GPE‐10 and compared with conventional LE systems. The oxidation stability of GPE‐10 was assessed through a constant‐voltage electrochemical floating test, where the leakage current was monitored during charging. As shown in Figure 7a, an increase in current up to 4.7 V demonstrates a wide electrochemical stability window (> 4.6 V) of GPE‐10 in NCM622 batteries, which is in agreement with the results from LSV curve (Figure 4b). This superior oxidative stability ensures its compatibility with Ni‐rich layered oxide cathodes operating at high potentials.

The rate capability of Li|GPE‐10|NCM622 and Li|LE|NCM622 batteries is compared in Figure 7b. At 0.1 *C*, both systems deliver comparable discharge capacities, indicating similar initial utilization of the cathode material. As the rate increases to 0.5 and 1.0 *C*, the GPE‐10 battery consistently delivers higher discharge capacities than the LE counterpart, demonstrating improved ion transport and interfacial kinetics. Even at 2.0 *C*, the GPE‐10 battery achieves 135.1 mAh g^−1^, nearly identical to that of the LE battery, while upon returning to 0.1 *C*, the discharge capacity recovers to 178.8 mAh g^−1^, substantially higher than that of the LE battery (156.7 mAh g^−1^). These results reveal that GPE‐10 enables faster reaction dynamics and excellent reversibility. As shown in Figure 7c, galvanostatic cycling at 0.5 *C* demonstrates the superior durability of the GPE‐based battery. Although the initial capacity of Li|GPE‐10|NCM622 is slightly lower than that of Li|LE|NCM622, it retains 130.0 mAh g^−1^ after 400 cycles, corresponding to 83.1% capacity retention and an average Coulombic efficiency of 99.8%. In contrast, the LE battery shows only 71.9% retention with a faster capacity fading rate of 0.112 mAh g^−1^ per cycle, compared to 0.065 mAh g^−1^ per cycle for GPE‐10 battery. The slower degradation rate of GPE‐10 battery highlights its enhanced interfacial stability and suppression of parasitic side reactions. The charge/discharge polarization behavior provides further evidence of this stabilization. Although the GPE‐10 battery exhibits a slightly larger initial polarization, its increase during cycling is much slower than that of the LE battery. After 400 cycles, the polarization voltage of the GPE‐10 battery remains at 0.20 V, smaller than the 0.28 V observed for the LE battery, confirming the mitigation of interfacial resistance growth (Figure 7d; Figure S15). The stable voltage profiles indicate that GPE‐10 effectively suppresses electrolyte decomposition and excessive SEI/cathode electrolyte interphase (CEI) accumulation, thereby maintaining the structural integrity of both electrodes during long‐term cycling. Collectively, these findings demonstrate that GPE‐10 not only provides excellent oxidation resistance at high voltages but also promotes stable electrode interfaces and improved reaction kinetics, enabling long‐lived and high‐performance Li||NCM622 batteries.

To further evaluate the applicability of GPE‐10 in high‐energy‐density systems, Li||NCM811 batteries with a high‐nickel cathode (LiNi_0.8_Co_0.1_Mn_0.1_O_2_ (NCM811)) were assembled. As shown in Figure S16a, at a high rate of 2.0 *C*, the Li|GPE‐10|NCM811 battery delivers nearly the same discharge capacity as the Li|LE|NCM811 battery, indicating comparable high‐rate capability. However, when the rate is subsequently reduced back to 0.1 *C*, the GPE‐10‐based battery exhibits a remarkably higher discharge capacity (170.8 mAh g^−1^) compared to the LE battery (159.7 mAh g^−1^), demonstrating improved reaction reversibility and more stable electrode interfaces. Long‐term cycling performance further highlights the stability advantage of the GPE system. As shown in Figure S16b, the Li|GPE‐10|NCM811 battery maintains a discharge capacity of 160.8 mAh g^−^^1^ after 200 cycles, corresponding to a capacity retention of 91.5%, while the LE counterpart retains only 82.5% of its initial capacity. The slower capacity fading of the GPE‐10 battery indicates that the polymer electrolyte effectively suppresses interfacial degradation and mitigates parasitic reactions, leading to more stable CEI formation during prolonged cycling. These findings are consistent with the results from Li||NCM622 systems and further confirm the universal capability of GPE‐10 to stabilize both the anode and cathode interfaces, thereby enabling durable cycling performance even in high‐voltage, Ni‐rich cathode configurations. Compared with recently reported polymer‐based electrolytes, GPE‐10 exhibits superior cycling performance (Figure 7e,f; Table S1), which indicates that GPE‐10 shows promising competitiveness for high‐voltage LMB applications.

### 2.8 Cathode Interfacial and Structural Stability Analysis

To further elucidate the interfacial chemistry and structural stability of the cathode in GPE‐based batteries, XPS, transmission electron microscopy (TEM), X‐ray diffraction (XRD), and inductively coupled plasma (ICP) analyses were conducted on the NCM622 electrodes after cycling.

**FIGURE 8:**
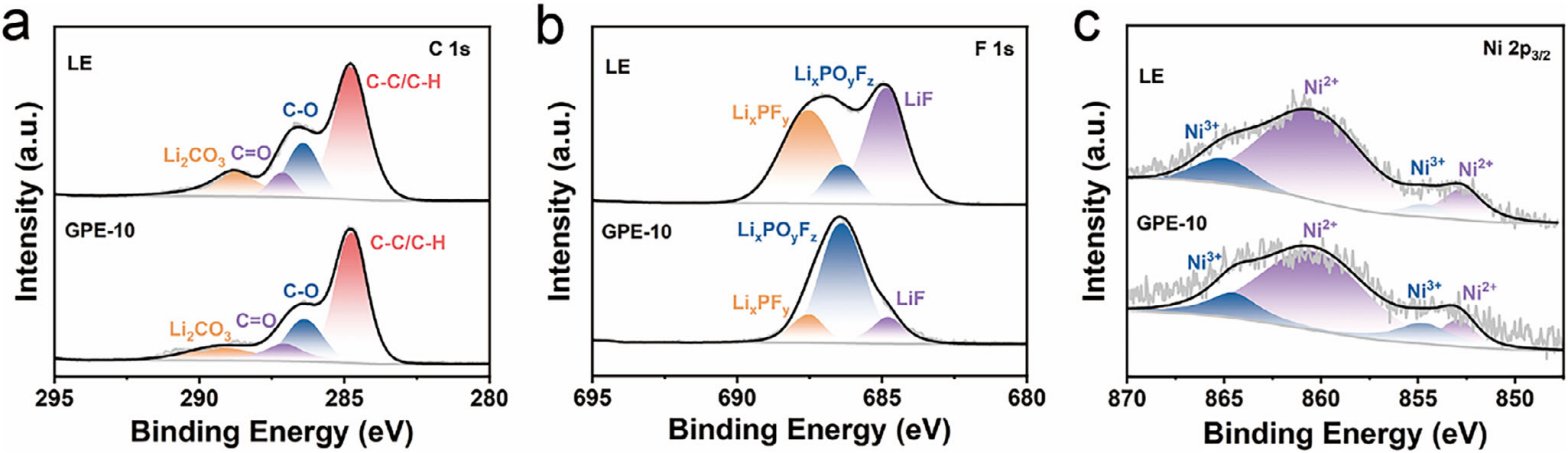
XPS spectra of (a) C 1s, (b) F 1s, and (c) Ni 2p_3/2_ of NCM622 cathode surface from the Li||NCM622 batteries with LE and GPE‐10 after 200 cycles.

As shown in Figure 8, the XPS spectra reveal distinct surface chemical differences between the cathodes from the GPE‐10 and LE batteries. In the C 1s spectra, the fraction of Li_2_CO_3_, a common by‐product from ester solvent decomposition, is significantly decreased on the cathode from the GPE‐10 battery (13.7%) compared with that from the LE battery (17.9%), suggesting suppressed oxidative decomposition of carbonate solvents. Similarly, in the F 1s spectra, the proportion of Li_x_PF_y_ species, which originate from LiPF_6_ degradation, decreases from 43.8% in the LE battery to 13.8% in the GPE‐10 battery, indicating improved oxidative stability of the electrolyte. In the Ni 2p_3/2_ spectra, peaks at 852.6 and 860.7 eV correspond to Ni^2+^, while those at 854.9 and 865.0 eV are assigned to Ni^3+^ [13]. The calculated Ni^2+^/Ni^3+^ ratio of 2.77 for the GPE‐10 cathode is markedly lower than that of 5.27 for the LE counterpart, signifying mitigated Li/Ni cation mixing [52]. Reduced cation disorder preserves the layered structure of NCM622 and contributes to enhanced structural reversibility during cycling. These XPS results collectively indicate that the GPE‐10 electrolyte effectively suppresses solvent and salt decomposition on the cathode, thereby stabilizing the interfacial chemistry and mitigating transition‐metal leaching. Further DFT calculations reveal that PEDMEP exhibits the highest occupied molecular orbital (HOMO) energy level (−7.48 eV) (Figure S18), indicating its higher tendency to be oxidized on the NCM622 surface than other electrolyte components. This preferential oxidation of PEDMEP at high potential promotes the formation of a robust CEI with a polymeric framework of high molecular weight, effectively mitigating transition‐metal dissolution and maintaining the structural integrity of the cathode surface [53]. The microstructure of the CEI on cycled NCM622 particles was further examined by TEM. As shown in Figure S19a, a thick and uneven CEI layer (ranging from 6.3 to 11.4 nm) is observed on the cathode from LE system after 35 cycles. In contrast, the NCM622 particle from the GPE‐10 system possesses a dense and uniform CEI layer with a thickness of approximately 2.9 nm (Figure S19b), which validates that GPE‐10 mitigates side reactions on the cathode surface. Collectively, the DFT calculations corroborate the XPS and TEM analyses, confirming that the GPE‐10 system contributes to a more stable interface.

The XRD analysis further supports these findings (Figure S20). After 200 cycles, the (003) diffraction peak of the cycled cathodes shifts slightly toward lower angles, indicative of lattice expansion associated with Li^+^ intercalation/deintercalation [54]. Notably, this shift is smaller for the cathode cycled in the GPE‐10 battery, implying that the polymer electrolyte effectively alleviates lattice strain and suppresses structural degradation. The *I*_003_/*I*_104_ intensity ratio, a key indicator of cation mixing and layered structure retention [55], remains nearly unchanged in the GPE‐10‐cycled cathode (1.90) compared with the pristine sample (1.93), while a significant decrease to 1.82 is observed in the LE‐cycled cathode. The higher *I*_003_/*I*_104_ ratio of the GPE‐10 sample confirms reduced Li/Ni disordering and enhanced phase stability.

To further verify interfacial stabilization, ICP measurements were conducted to quantify the concentrations of dissolved transition metals (Ni, Co, and Mn) ions in the electrolytes after cycling (Figure S21). The GPE‐10 system exhibits markedly lower transition‐metal dissolution compared with the LE system, underscoring its strong capability to suppress cathode corrosion and metal ion migration. Collectively, the XPS, XRD, and ICP results provide compelling evidence that the LiNO_3_‐based GPE‐10 electrolyte effectively stabilizes the NCM622 cathode by suppressing electrolyte decomposition, mitigating cation mixing, preserving the layered structure, and reducing transition‐metal dissolution. These synergistic effects ensure superior interfacial stability and long‐term cycling durability of high‐voltage layered oxide cathodes.

## 3 Conclusion

In summary, a phosphorus‐containing multifunctional monomer (EDMEP) was successfully utilized to enable LiNO_3_ dissolution within a conventional ester‐type electrolyte system and in situ formation of a flame‐retardant GPE. The formed GPE effectively confines liquid components, and possesses exceptional flame‐retardant properties, enhancing both the electrochemical and safety performance of the batteries. The Li|GPE‐10|Li symmetric cell reveals that GPE‐10 can promote the formation of Li_3_N‐LiF‐rich SEI layer on the Li metal anode, facilitating Li^+^ transport and promoting dense and smooth Li deposition. When used in LMBs with high‐voltage cathodes (NCM622 and NCM811), the GPE‐10 system exhibits excellent rate performance and outstanding long‐term cycling stability, outperforming its LE counterparts. Mechanism analyses confirm that the GPE‐10 significantly suppresses electrolyte decomposition, transition‐metal dissolution, and Li/Ni cation mixing, thus ensuring the structural integrity of layered oxide cathodes. This work presents a rational electrolyte design concept that unifies LiNO_3_ solvation, interfacial protection, and intrinsic flame retardancy, paving the way toward high‐energy‐density and intrinsically safe lithium metal batteries.

## Conflicts of Interest

The authors declare no conflicts of interest.

## Supporting information




**Supporting File 1**: advs74169‐sup‐0001‐SuppMat.docx.

**Supporting File 2**: advs74169‐sup‐0002‐VideosS1‐S6.zip.

## Data Availability

The data that support the findings of this study are available in the supplementary material of this article.
