# Supplementary material for: Multifunctional Phosphate Monomer Enabling LiNO3 Solvation and In Situ Formation of Flame‐Retardant Gel Polymer Electrolyte for High‐Voltage Lithium Metal Batteries
Source: Adv Sci (Weinh). 2026 Jan 31;13(20):e24165. doi: 10.1002/advs.202524165 (PMC13067821; doi:10.1002/advs.202524165)
Supplement: Supplementary file 1 — Supporting File 1: advs74169‐sup‐0001‐SuppMat.docx. [file ADVS-13-e24165-s001.docx]

Supporting Information

**Multifunctional Phosphate Monomer Enabling LiNO_3_ Solvation and In-situ Formation of Flame-retardant Gel Polymer Electrolyte for High-voltage Lithium Metal Batteries**

*Lijun Ma, Lu Lu, Tianqi Xiang, Yaxin Wang, Jianjun Zhou*,* *and Lin Li**

L. Ma, L. Lu, T. Xiang, Y. Wang, J. Zhou, L. Li

College of Chemistry, Beijing Normal University, Beijing 100875, China

E-mail: lilinll@bnu.edu.cn; pla_zjj@bnu.edu.cn

L. Li

College of Textiles & Clothing, Qingdao University, Qingdao 266071, China

**Experimental Section**

**Materials**

Ethyl dichlorophosphate, Triethylamine (Et_3_N), Dichloromethane (DCM), 2-hydroxyethyl methacrylate, Lithium nitrate (LiNO_3_), azobisisobutyronitrile (AIBN) and *N*-methyl-2-pyrrolidone (NMP) were purchased from Macklin. The LiNi_0.6_Co_0.2_Mn_0.2_O_2_ (NCM622), LiNi_0.8_Co_0.1_Mn_0.1_O_2_ (NCM811), conductive graphite, acetylene black and poly(vinylidene fluoride) (PVDF) were commercially obtained from Kejing Zhida Technology Co., Ltd, China. Ester-type liquid electrolyte (LE, 1.0 M LiPF_6_ in ethylene carbonate (EC)/ethyl methyl carbonate (EMC) = 3: 7 by volume with 5 wt% fluoroethylene carbonate (FEC)) was purchased from Suzhou Dodo Chem. Polyethylene (PE) separator (14 μm) was kindly supplied by SEMCORP, China.

**Sample Preparation**

Preparing ethyl di(2-(methacryloyloxy) ethyl) phosphate (EDMEP): 2-hydroxyethyl methacrylate (2.776 g, 21 mmol, 2.1 eq.) and Et_3_N (2.453 g, 24 mmol, 2.4 eq.) were dissolved in DCM (20 mL). And ethyl dichlorophosphate solution (1.715 g, 10 mmol, 1 eq. dissolved in DCM (10 mL)) was added at 0 ℃. After stirring at room temperature for 12 h, the mixture was filtered and then washed by DCM, deionized water, hydrochloric acid (10%) and saturated NaCl solution. The organic layer was dried by anhydrous Na_2_SO_4_. The final product was obtained after removal of the solvent by rotary evaporation. ^1^H NMR (400 MHz, Chloroform-*d*, δ): 6.08 (d, *J* = 12.4 Hz, 2H), 5.53 (d, *J* = 8.4 Hz, 2H), 4.41–4.25 (m, 4H), 4.24–4.14 (m, 4H), 4.05 (q, *J* = 7.6 Hz, 2H), 1.86 (s, 6H), 1.25 (t, *J* = 7.16 Hz, 3H); ^31^P NMR (400 MHz, Chloroform-*d*, δ): −0.71.

Preparing GPE: EDMEP was weighed and placed into a 5 mL transparent reagent bottle, followed by dissolution with LE. LiNO_3_ was weighed and added to the prepared mixture, followed by stirring at 60 °C until complete dissolution. Then, AIBN was added as an initiator in the ratio of 1 wt% of EDMEP to obtain transparent precursor LE. The precursor LE was heated at 65 °C for 8 h to initiate the free radical polymerization reaction. GPE was obtained.

Preparing polymer matrix (PEDMEP): To obtain the PEDMEP, the GPE-10 was washed with dimethyl carbonate and then centrifuged at 3500 rmp for 3min. The supernatant liquid was removed, and then the washing and centrifugation steps were repeated three times. Finally, the PEDMEP was dried in vacuum oven at 80 ℃ overnight.

Preparing cathode: NCM622, conductive graphite, acetylene black and PVDF were mixed in a mass ratio of 85: 5: 5: 5, in which PVDF was added in NMP solution with the concentration of 5 wt%. The mixture was grinded with appropriate NMP to homogenize the slurry. The slurry was casting on carbon-coated aluminum foil and dried in drying oven for 3 h at 65 ℃, then 24 h in vacuum oven at 120 ℃. The NCM622 cathode was punched into disks with the diameter of 10 mm. The mass load of NCM622 was approximately 4 mg cm^−2^. The NCM811 cathode was also prepared with the same procedures.

**Material Characterization**

JNM-ECZ600R was used to attain proton, phosphorus and fluorine nuclear magnetic resonance spectroscopy (^1^H NMR, ^31^P NMR and ^19^F NMR spectra) in chloroform-*d* and DMSO-*d*_6_, respectively. Fourier transform infrared spectroscopy (FTIR) (Bruker VERTEX 70) was used to study functional groups of EDMEP and GPE-10 at 400–4000 cm^−1^. The morphology was characterized by field-emission scanning electron microscopy (SEM, SU-8010, Hitachi). X-ray photoelectron spectroscopy (XPS) analysis was conducted using an ESCALAB 250 Xi analyzer integrated with monochromic Al Kα (1486.6 eV) X-ray beam radiation (15 kV and 20 mA) and binding energy was calibrated *vs.* carbon (C 1s = 284.8 eV). Inductively coupled plasma (ICP) was recorded on Thermo ICAP6300. X-ray diffraction (XRD) data was obtained using a D/MAX-3C X-ray diffraction meter with Cu Kα (λ = 1.5406 Å) radiation. Raman spectroscopy was conducted using a Thermo DXR machine at room temperature with a 532 nm laser. The thermal stability was studied using a thermal analysis instrument (STA449 F5, Mettler Toledo) in the temperature range of 25–600 ℃ under a N_2_ atmosphere at a heating rate of 20 ℃ min^−1^. Transmission electron microscopy (TEM, Thermo Fisher FEI Tecnai TF20) was used to identify the CEI thickness on the cycled NCM622 cathodes.

**Combustion Test**

Following the drop-casting of 0.15 g of baseline LE or precursor LE onto a 19 mm glass fiber separator to achieve complete infiltration, both the infiltrated separators and the pure gels were evaluated in ignition tests.

**Cell Assembly**

CR2032 coin cells were assembled to evaluate the GPE-10. Li||NCM622 batteries were assembled with the following procedures. In a positive battery shell, NCM622 cathode, 25 μL LE or precursor LE, a piece of PE separator (Ф = 19 mm), another 25 μL LE or precursor LE, Li foil (Ф = 15.6 mm), stainless steel (SS) plate, spring plate and the negative battery shell were put on sequentially and sealed. For the batteries filled with precursor LE, they were heated at 65 ℃ for 8 h to prepare Li|GPE-10|NCM622 batteries with the in-situ formed GPE. Li||NCM811, Li||Li, SS||SS, Li||SS and Li||Cu batteries were also prepared using LE or precursor LE with the same procedures.

**Electrochemical Characterization**

Electrochemical impedance spectroscopy (EIS) was measured using an electrochemical workstation (Interface 1010E, Gamry) at 25 ℃ and within a frequency range of 10^6^–10^−1^Hz. The ionic conductivity was calculated using the resistance from SS||SS battery according to the following equation:

$\sigma=L/(R\times S)$ (S1)

where *L* is the thickness of GPE, *R* is the resistance, and *S* is the contact area. The activity energy of Li^+^ transport can be calculated by the following equation:

σ$=A exp(-\frac{E_{a}}{RT})$ (S2)

where *A* is the pre-exponential factor, *E_a_* is the activation energy of Li^+^ transport, *R* is the ideal gas constant, and *T* is temperature. The Li^+^ transference number (*t*_Li⁺_) in Li||Li symmetric cell was determined using chronoamperometry with a direct current pulse of 10 mV and EIS tests at 25 ℃. The *t*_Li⁺_ was calculated from the following equation:

$t_{{Li}^{+}}=\frac{I_{s}(\Delta V-I_{0}R_{0})}{I_{0}(\Delta V-I_{s}R_{s})}$ (S3)

where *R_0_* and *R_s_* represent the impedance values before and after polarization testing, respectively, while *I_0_* and *I_s_* denote the initial current and steady-state current, correspondingly. Δ*V* represents the externally applied voltage during the polarization measurement.

The electrochemical stability window was characterized by the linear sweep voltammetry (LSV) of Li||SS cell at 25 ℃, with a scanning rate of 0.1 mV s^−1^. The exchange current density was determined from Tafel plot of Li||Li cell obtained at a scan rate of 0.2 mV s^−1^ and within a voltage range of -0.2–0.2V. The cyclic voltammetry (CV) curve of Li||Cu cell was measured between 0 and 3.0 V at 5 mV s^−1^ scanning rate. The assembled cells were tested on a battery testing system (CT2001A LANHE) at 25 ℃. Li||Li symmetric cells were cycled at 0.2 mA cm^−2^ and 0.2 mAh cm^−2^ or 0.5 mA cm^−2^ and 0.5 mAh cm^−2^. The Li||NCM622 and Li||NCM811 batteries were tested within a voltage range of 2.8–4.3 V. EIS was performed on Li||Li symmetric cells after various cycling durations (corresponding to different numbers of cycles) at 0.2 mA cm^−2^ and 0.2 mAh cm^−2^. Prior to each EIS measurement, the cells were fully discharged to ensure that all impedance data were collected under a consistent state of charge.

**Theoretical Calculation**

Density functional theory (DFT) was employed to optimize the structures and calculate molecular orbital energy, electrostatic potential (ESP) and the binding energy (*E_b_*), using the B3LYP functional method and the 6–31+G (d, p) basis set. All calculations were performed using the Gaussian 16 program package. The *E_b_* of substances A and B was calculated using the following equation:

$E_{b}=E_{A/B}-E_{A}-E_{B}$ (S4)

where *E_A/B_* is the energy of the interaction system of A and B, and *E_A_* and *E_B_* are the energies of A and B, respectively.

Molecular dynamics (MD) simulations were conducted with the Materials Studio 2023 program. The interactions between solvents and Li^+^ were described by COMPASS Ⅲ force field, and the charges of all particles were given by the force field. To determine the electrolyte structure, the amorphous cell (46.6Å × 46.6Å × 46.6Å) was constructed using the optimal molecules and ions. The LE electrolyte model contained 260 EC, 400 EMC, 36 FEC, 60 Li^+^ and 60 PF_6_^−^ molecules. The EDMEP/LiNO_3_ LE electrolyte model contained 270 EC, 408 EMC, 26 EDMEP, 42 FEC, 73 Li^+^, 60 PF_6_^−^ and 13 NO_3_^−^ molecules. The cell parameters were firstly optimized by Forcite module using COMPASS Ⅲ forcefield. And then the system was subjected to an annealing procedure by cycling the temperature between 300 K and 500 K for 20 cycles to ensure proper equilibration and escape from local energy minima. Subsequently, the system was equilibrated at 298 K in the NPT ensemble for 1 ns, with a pressure of 10^−4^ GPa. The temperature and pressure were controlled by Berendsen thermostat and Berendsen barostat algorithm, respectively. Finally, a MD run in the NVT ensemble was performed for 1 ns at 298 K using NHL thermostat for equilibrium, and a following 2 ns NVT simulation was used to obtain the data.


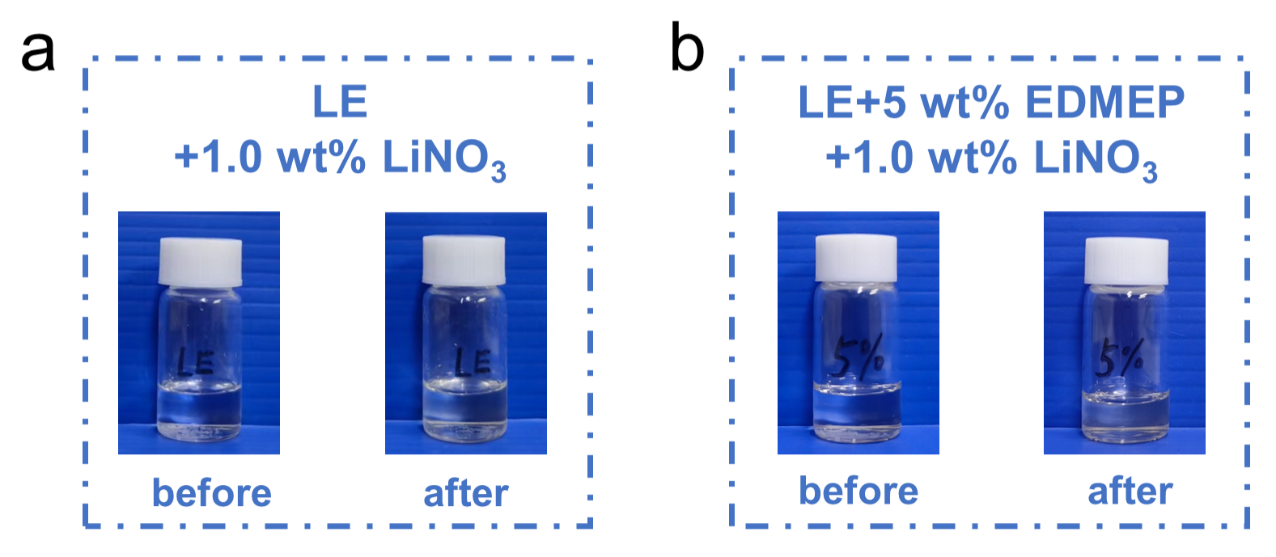


**Figure S1.** Optical images of (a) the baseline LE and (b) the electrolyte containing 5.0 wt% EDMEP, before and after the dissolution of 1.0 wt% LiNO_3_.


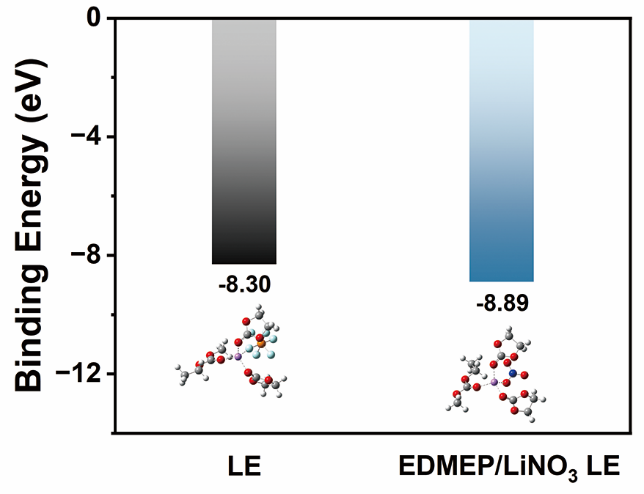


**Figure S2.** The binding energies of the first Li^+^ solvation structures for LE and EDMEP/LiNO_3_ contained LE system.


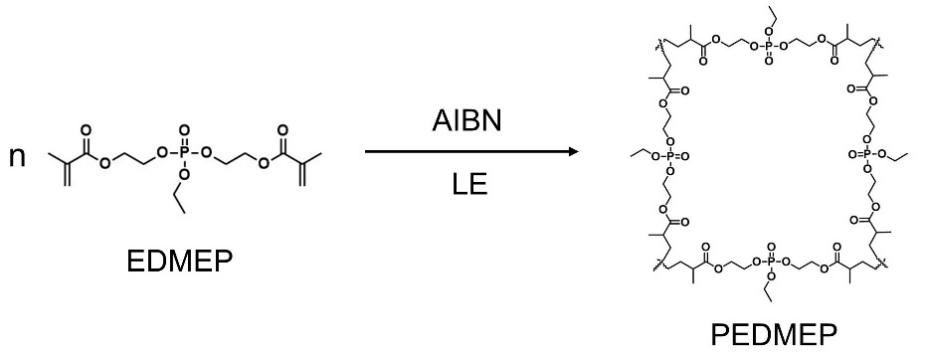


**Figure S3.** The free radical polymerization of EDMEP.


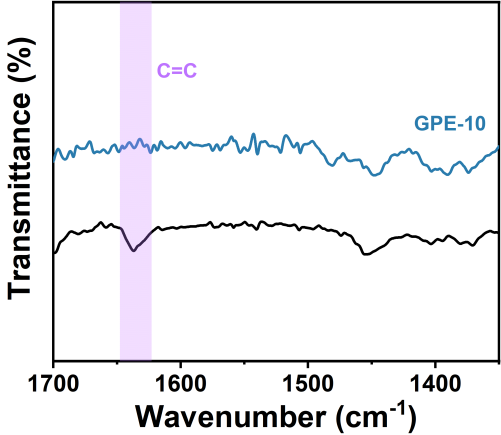


**Figure S4.** FTIR profiles of EDMEP and GPE-10.


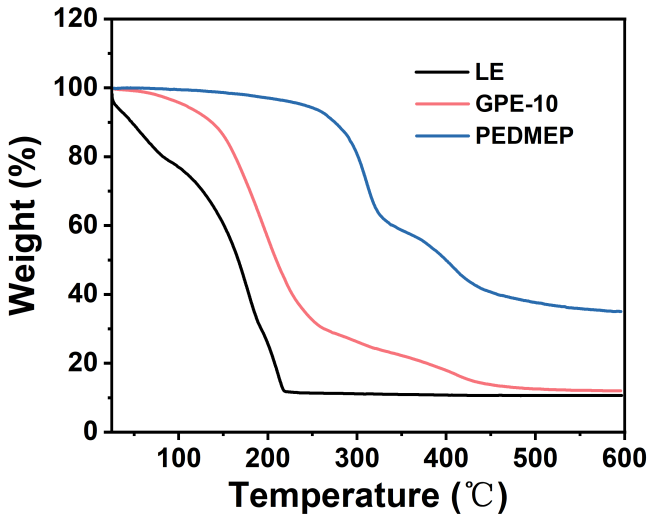


**Figure S5.** TGA curves of LE, GPE-10, and PEDMEP.


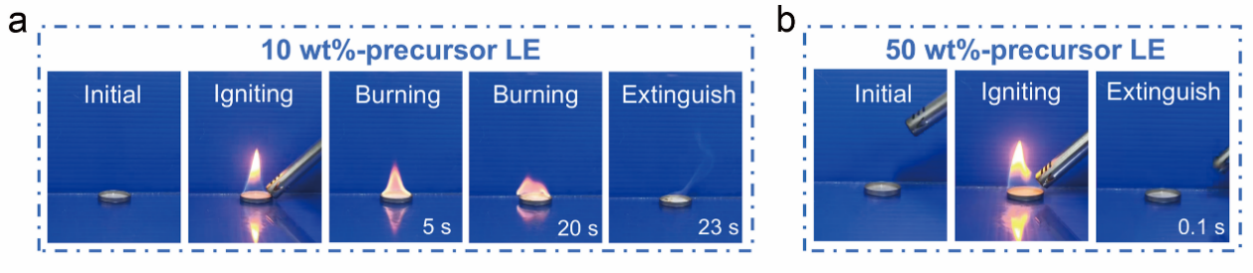


**Figure S6.** The combustion experiments of precursor LEs containing (a) 10.0 wt% and (b) 50.0 wt% EDMEP.


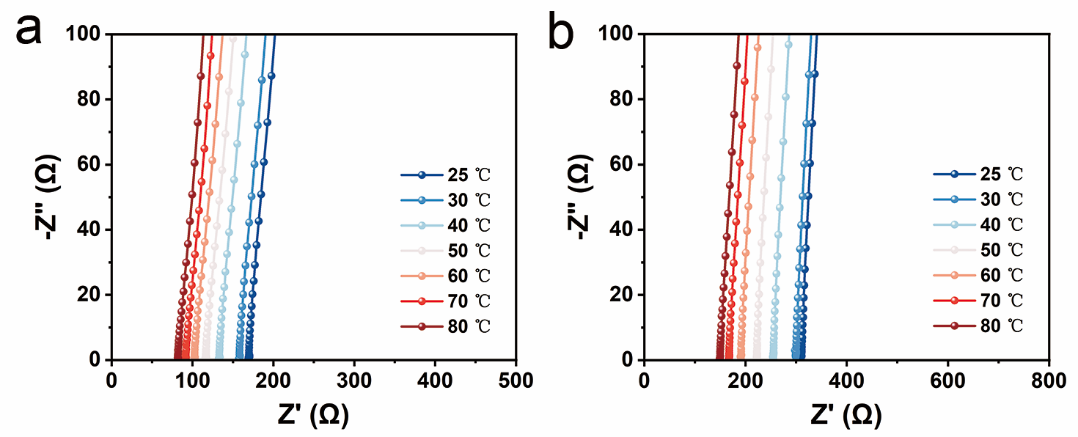


**Figure S7.** Temperature-dependent impedance spectra of the (a) SS|LE|SS and (b) SS|GPE-10|SS cells.


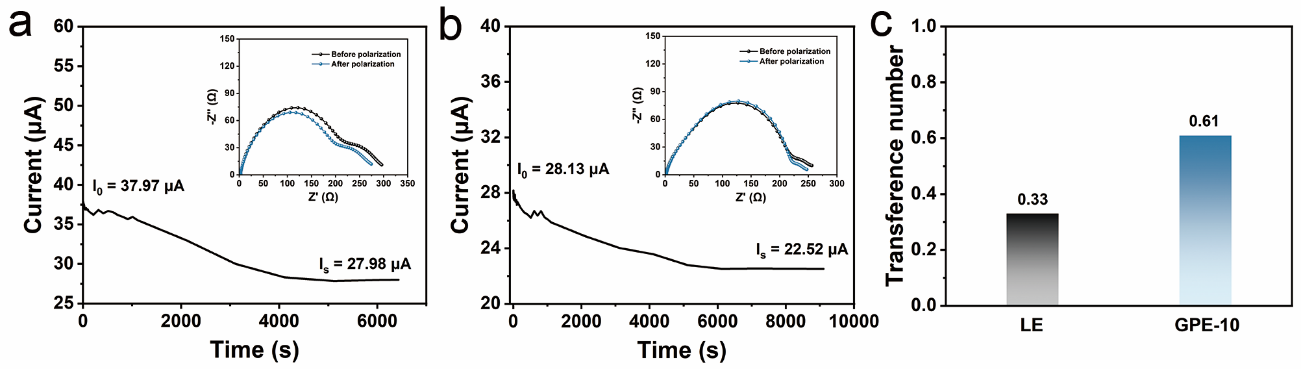


**Figure S8.** Polarization curves as well as the initial and steady state impedance diagrams of (a) Li|LE|Li and (b) Li|GPE-10|Li cells. (c) The Li^+^ transference number of LE and GPE-10.


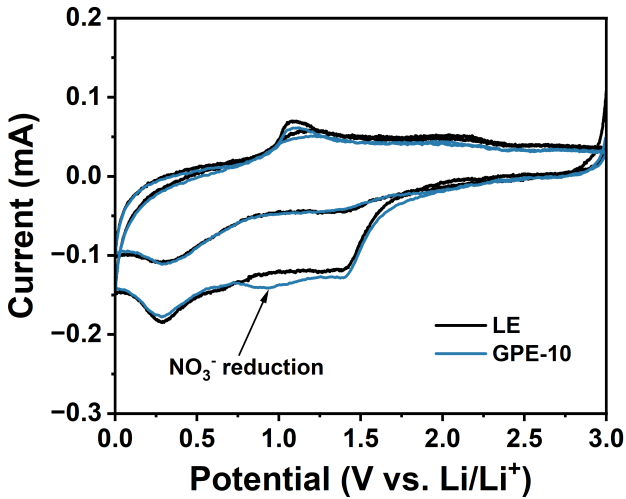


**Figure S9.** CV curves of Li||Cu cells for the LE and GPE-10.


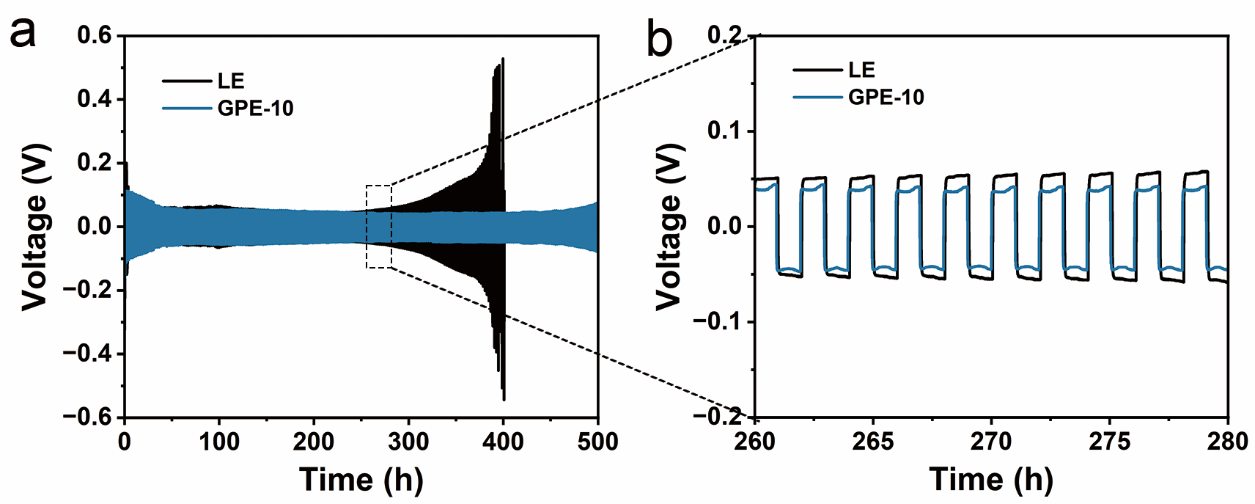


**Figure S10.** (a) Voltage profiles of Li||Li symmetric cells at 0.5 mA cm^−2^ and 0.5 mAh cm^−2^. (b) Enlarged voltage profiles.


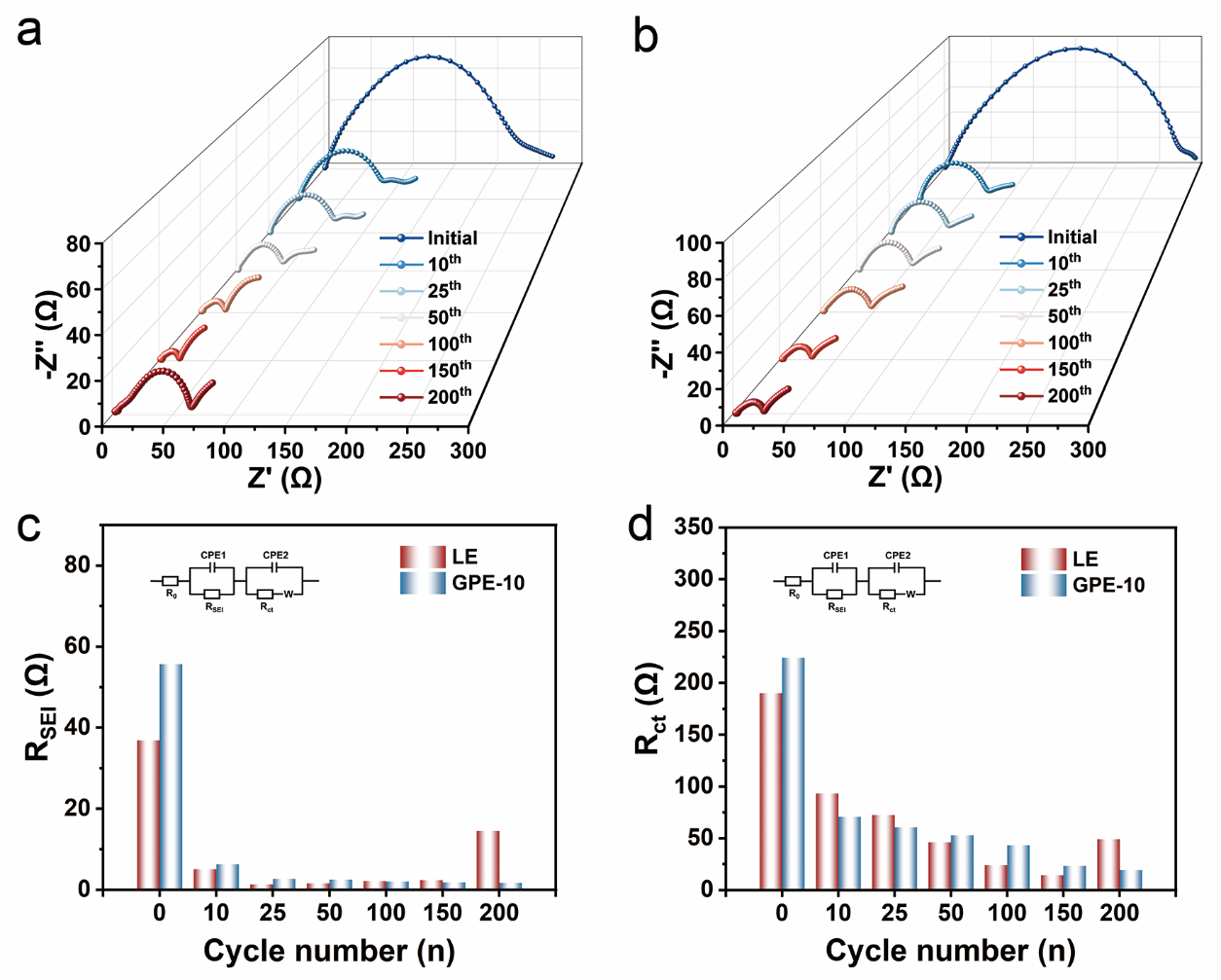


**Figure S11.** Nyquist plots of Li||Li cells with (a) LE and (b) GPE-10 cycling at 0.2 mA cm^−2^ and 0.2 mAh cm^−2^. The fitted (c) *R*_SEI_ and (d) *R*_ct_ values (inset: the corresponding equivalent circuit).


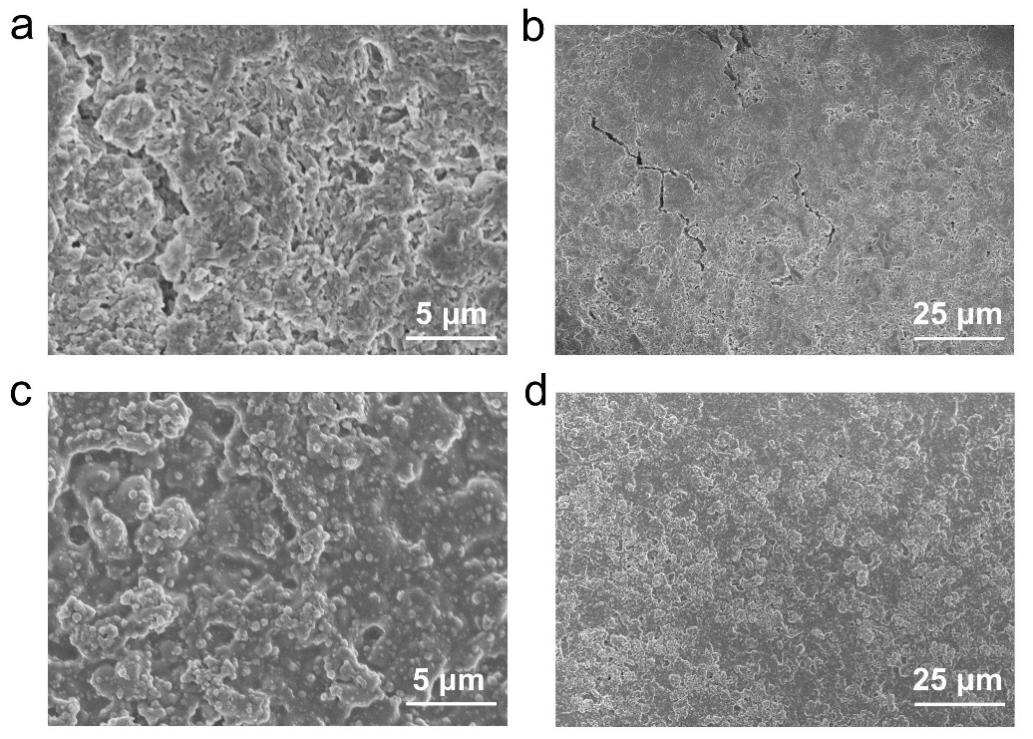


**Figure S12.** The surface morphology of Li anode from (a, b) Li||LE||Li and (c, d) Li||GPE-10||Li cells after 100 cycles.


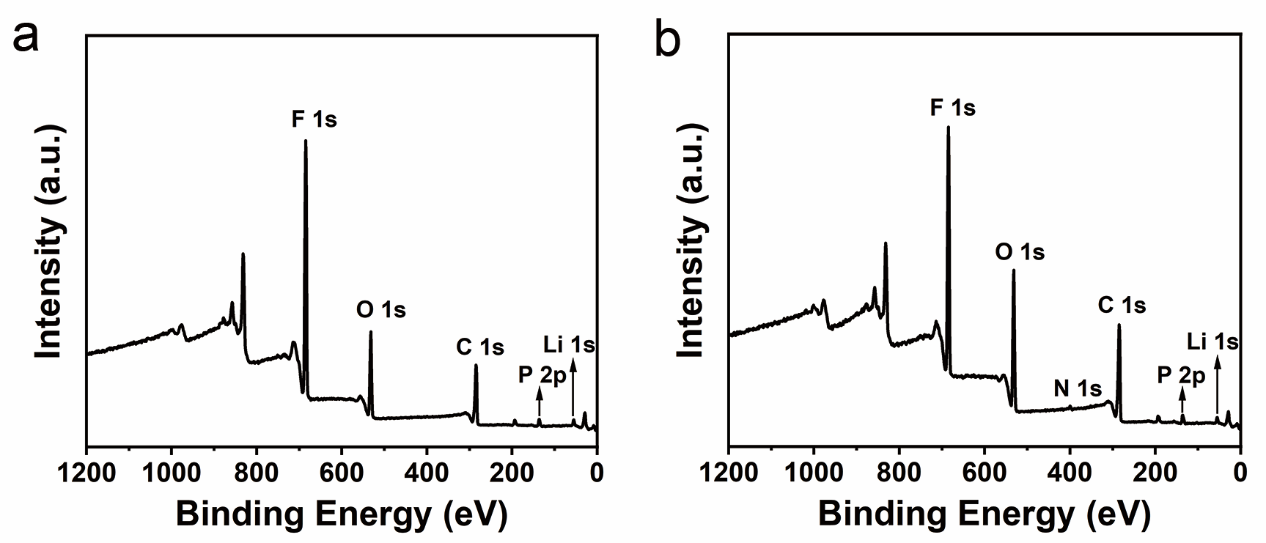


**Figure S13.** XPS survey spectra of Li anode surface in the Li||Li cells with (a) LE and (b) GPE-10 after 100 cycles.


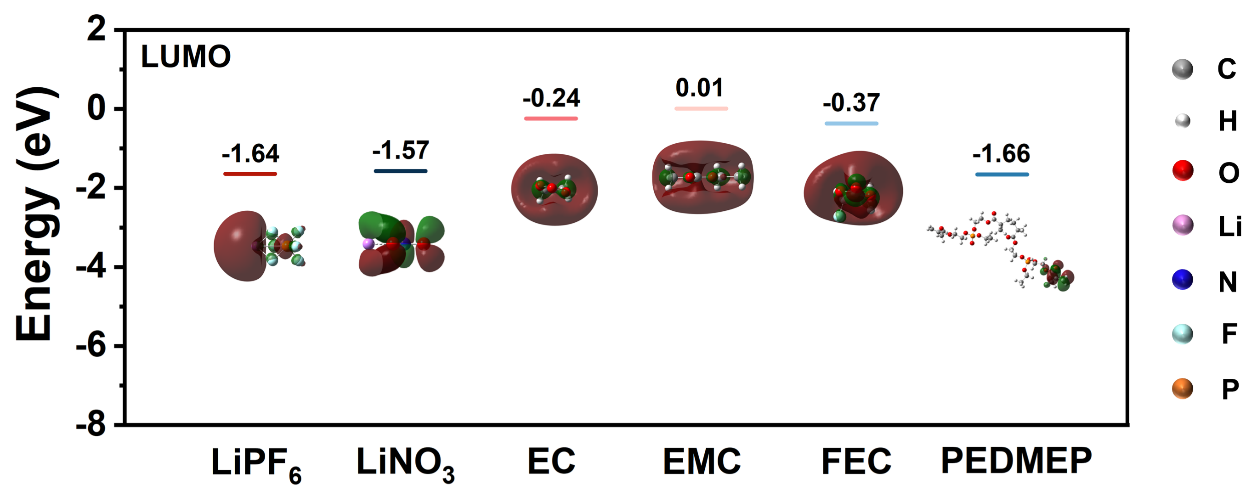


**Figure S14.** LUMO of the components in LE, LiNO_3_ and PEDMEP.


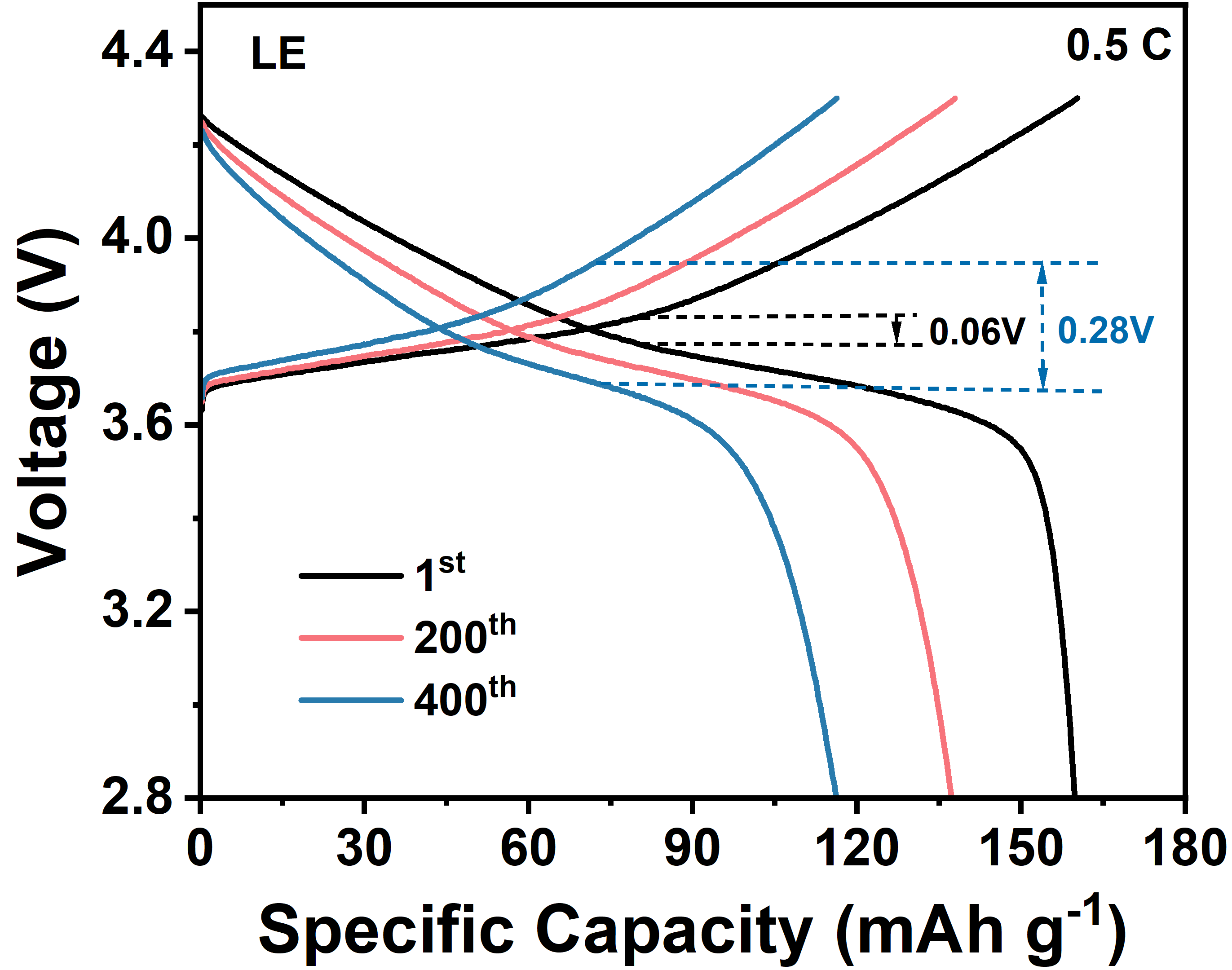


**Figure S15.** Charge-discharge profiles of Li|LE|NCM622 battery.


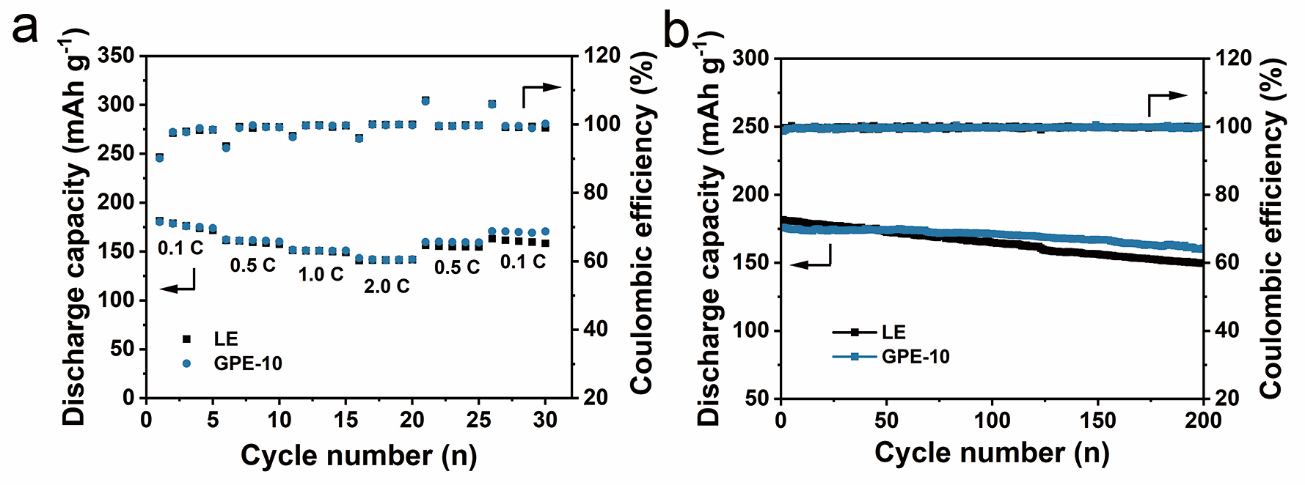


**Figure S16.** (a) *C*-rate and (b) cycling performances of Li||NCM811 batteries.


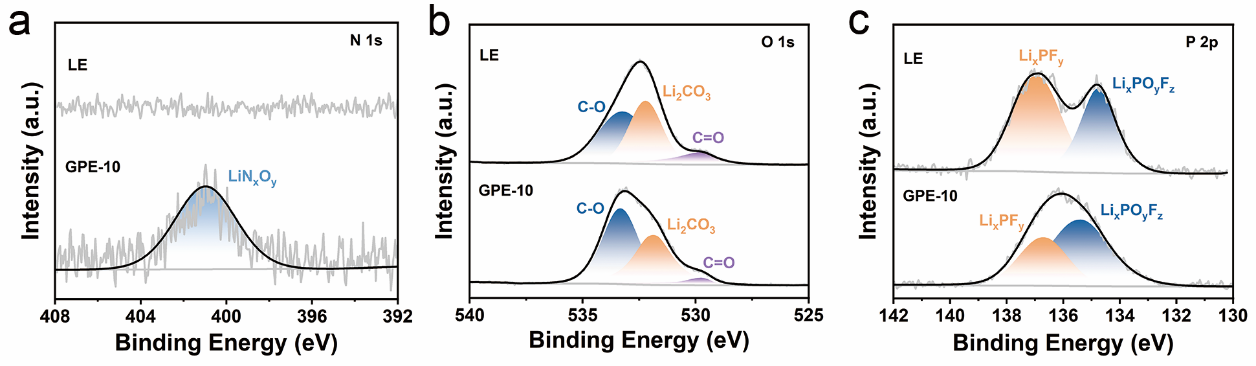


**Figure S17.** XPS spectra of (a) N 1s, (b) O 1s and (c) P 2p of NCM622 cathode surface from the Li||NCM622 batteries with LE and GPE-10 after 200 cycles.


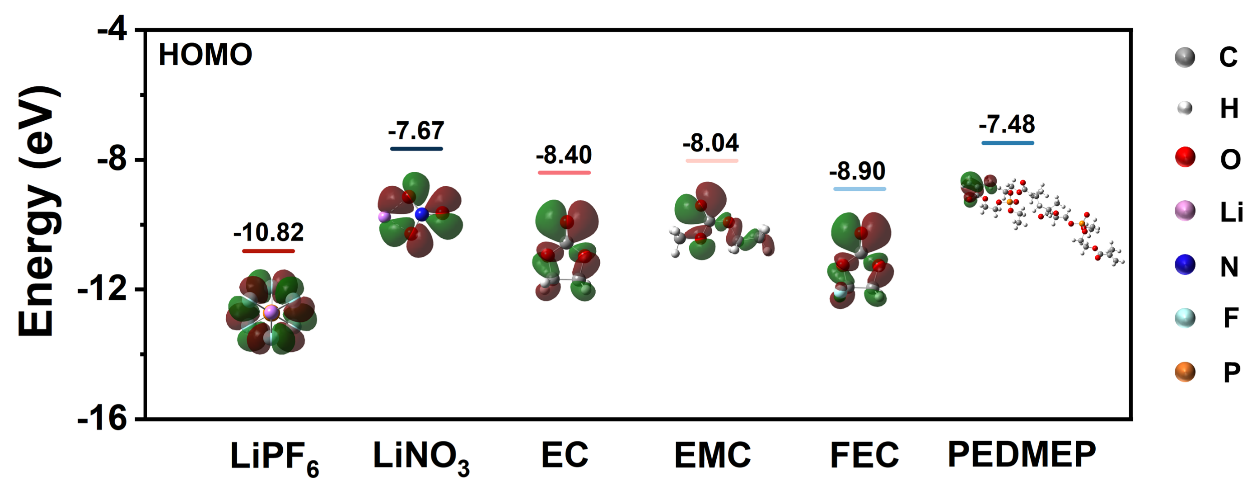


**Figure S18.** HOMO of the components in LE, LiNO_3_ and PEDMEP.


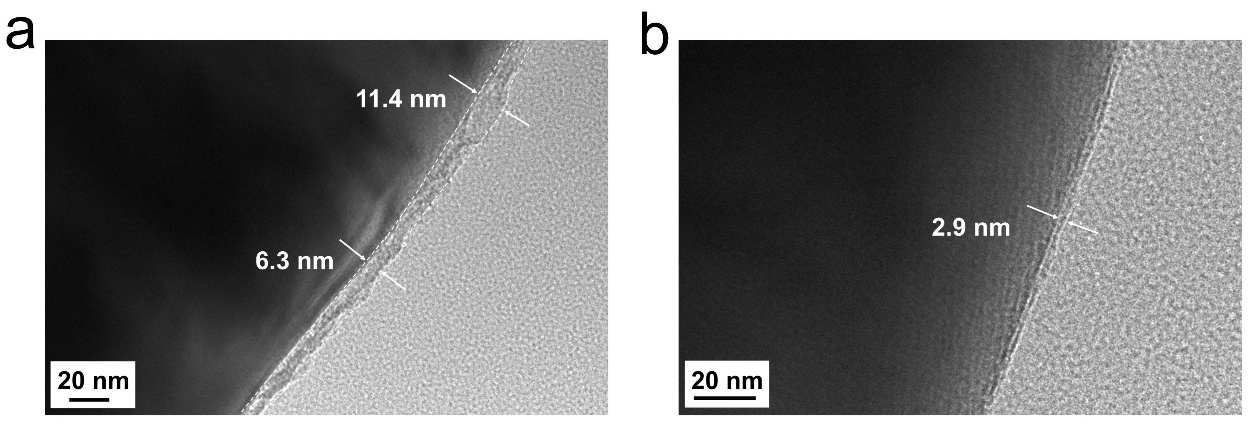


**Figure S19.** TEM images of NCM622 particles obtained from (a) Li|LE|NCM622 and (b) Li|GPE-10|NCM622 batteries after 35 cycles.


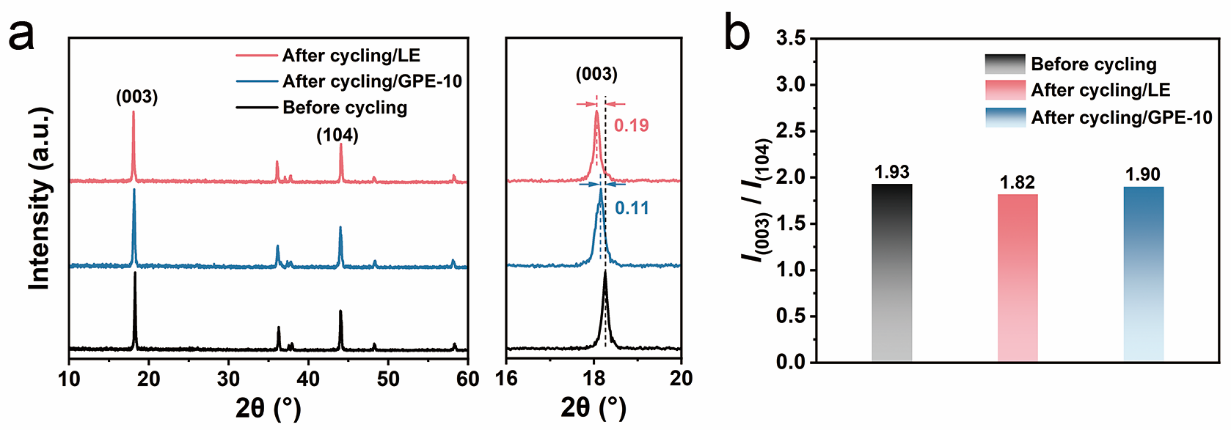


**Figure S20.** (a) XRD spectra and local magnification of (003) diffraction peak of the NCM622 cathodes before and after 200 cycles with LE and GPE-10. (b) The corresponding ratios of *I*_003_/*I*_104_.


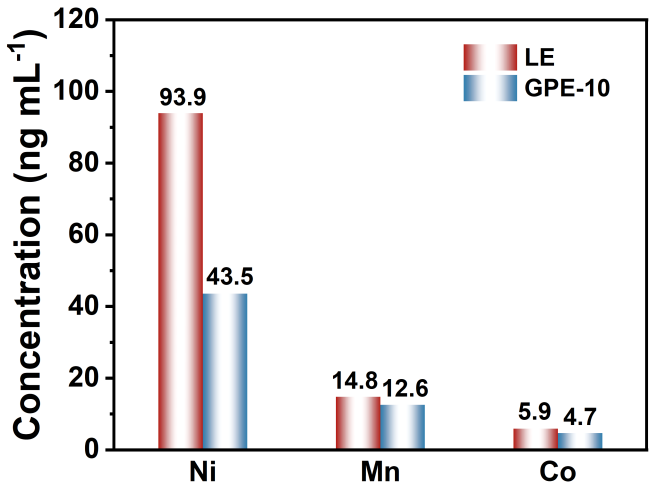


**Figure S21.** Comparison of the solubility of transitional metal ions in NCM622 cathodes after 200 cycles through ICP analysis.

**Table S1.** Comparison of cycling performances of GPE-10 with other reported polymer electrolytes in Li||NCM batteries.

| **Ref.** | **Cathode** | **Electrolyte** | **Rate [*C*]** | **Cycle number** | **Capacity retention [%]** |
| --- | --- | --- | --- | --- | --- |
| This work | NCM622 | GPE-10 | 0.5 | 400 | 83.1 |
| This work | NCM811 | GPE-10 | 0.5 | 200 | 91.5 |
| S1 | NCM622 | IWSWN-SPE | 0.5 | 300 | 73.5 |
| S2 | NCM622 | GPE-7.5 | 0.5 | 400 | 82.1 |
| S3 | NCM622 | MOFLi/MSLi QSE | 1.0 | 200 | 70.0 |
| S4 | NCM622 | LNO/DMAA GPE | 1.0 | 400 | 80.1 |
| S5 | NCM622 | PIQSE | 0.2 | 200 | 85.0 |
| S6 | NCM622 | *c*-PHAP-GPE | 0.5 | 500 | 81.9 |
| S7 | NCM622 | BDFPE | 0.5 | 150 | 84.3 |
| S8 | NCM622 | FPH-Li | 1.0 | 300 | 81.5 |
| S9 | NCM811 | GPE 15% | 0.2 | 150 | 76.5 |
| S10 | NCM811 | PPUM-PE | 0.5 | 250 | 85.5 |
| S11 | NCM811 | NFAA-DGPE | 0.5 | 300 | 84.0 |
| S12 | NCM811 | BADBP-PHL | 0.5 | 220 | 82.4 |
| S13 | NCM811 | CSE | 0.5 | 400 | 61.0 |
| S14 | NCM811 | FRSPE | 0.5 | 200 | 77.0 |
| S15 | NCM811 | P(VEC-TFPMA) | 0.2 | 100 | 89.7 |
| S16 | NCM811 | SPE | 0.5 | 200 | 71.0 |
| S17 | NCM811 | PETEA-TCGG-PAN | 0.5 | 150 | 91.0 |

**References**

[S1] J. Chen, Chang. He, Xu. Peng, J. Li, X. Xu, Y. Zhou, J. Shen, J. Sun, Y. Li, T. Zhao, Puzzle-like Molecular Assembly of Nonflammable Solid-state Polymer Electrolytes for Safe and High-voltage Lithium Metal Batteries, *Nat. Commun.* **2025**, *16*, No. 8494. <https://doi.org/10.1038/s41467-025-63439-6>.

[S2] T.Q. Xiang, Z.H. Zhang, H. Huo, J.J. Zhou, L. Li, Upgrading Ester-based Electrolyte with LiNO_3_ and GPE to Realize Dendrite-free Lithium Deposition, *Small* **2026**, *22*, No. e11417. <https://doi.org/10.1002/smll.202511417>.

[S3] Z. Chen, W. Zhao, Q. Liu, Y. Xu, Q. Wang, J. Lin, H.B. Wu, Janus Quasi‑solid Electrolyte Membranes with Asymmetric Porous Structure for High‑performance Lithium‑metal Batteries, *Nano-Micro Lett.* **2024**, *16*, No. 114. <https://doi.org/10.1007/s40820-024-01325-4>.

[S4] C. Jing, K. Dai, D. Liu, W. Wang, L. Chen, C. Zhang, W. Wei, Crosslinked Solubilizer Enables Nitrate-enriched Carbonate Polymer Electrolytes for Stable, High-voltage Lithium Metal Batteries, *Sci. Bull.* **2024**, *69*, 209-217. <https://doi.org/10.1016/j.scib.2023.11.039>.

[S5] M.N. Li, F. Shen, K.M. Wang, Z. Zhang, X.G. Han, Non-flammable and Stable Phosphate Quasi-solid Electrolyte with Low Salt Concentration for Lithium Metal Batteries, *Rare Met.* **2025**, *44*, 3761-3771. <https://doi.org/10.1007/s12598-025-03239-5>.

[S6] S.X. Jia, T.Q. Xiang, Z.H. Zhang, J.J. Zhou, L. Li, In-situ Forming Flame-retardant Gel Polymer Electrolyte through Ritter Reaction: An Innovative Strategy for Enhancing the Safety of Lithium Metal Battery, *Chem. Eng. J.* **2025**, *503*, No. 158563. <https://doi.org/10.1016/j.cej.2024.158563>.

[S7] Q. Ma, S. Fu, A.J. Wu, Q. Deng, W.D. Li, D. Yue, B. Zhang, X.W. Wu, Z.L. Wang, Y.G. Guo, Designing Bidirectionally Functional Polymer Electrolytes for Stable Solid Lithium Metal Batteries, *Adv. Energy Mater.* **2023**, *13*, No. 2203892. <https://doi.org/10.1002/aenm.202203892>.

[S8] P. Zhai, Z. Yang, Y. Wei, X. Guo, Y. Gong, Two-dimensional Fluorinated Graphene Reinforced Solid Polymer Electrolytes for High-performance Solid-state Lithium Batteries, *Adv. Energy Mater.* **2022**, *12*, No. 2200967. <https://doi.org/10.1002/aenm.202200967>.

[S9] H. Zhang, Z. Ni, Z. Wang, Y. Li, S. Liu, J. Liu, C. Yang, S. Xiong, B. Xi, J. Feng, Manipulating Competitive Li^+^ Coordination of F-rich Polymer and High Flash Point Glyme Electrolyte Enabling High Rate and Intrinsically Safe Quasi-solid-state Li Metal Batteries, *Angew. Chem. Int. Ed.* **2025**, *64*, No. e202508281. <https://doi.org/10.1002/anie.202508281>.

[S10] Y. Wang, S. Zhang, Z. Chen, H. Zhang, F. Tian, J. Wang, Y. Zhu, G. Yang, Z. Li, G. Cui, Long-life Lithium Metal Batteries Enabled by In Situ Solidified Polyphosphoester-based Electrolyte, *Adv. Mater.* **2026**, *38*, No. e14210. <https://doi.org/10.1002/adma.202514210>.

[S11] Y. Shi, X. Miao, G. Zhao, S. Xiao, Z. Guo, Constructing the Non-flammable Anion Acceptor-containing Deep Eutectics-based Gel Polymer Electrolyte for High-performance Li-metal Batteries, *Adv. Funct. Mater.* **2025**, No. e24608. <https://doi.org/10.1002/adfm.202524608>.

[S12] K. Chen, A. Hu, W. Yang, Y. Li, Z.W. Seh, F. Li, J. Long, S. Chen, Symmetrical Molecular Topology Enables Ultrathin Solid Polymer Electrolytes for Stable Lithium-metal Batteries, *Adv. Funct. Mater.* **2026**, *36*, No. e13143. <https://doi.org/10.1002/adfm.202513143>.

[S13] A.G. Nguyen, M.H. Lee, J. Kim, C.J. Park, Construction of a High‑performance Composite Solid Electrolyte through In‑situ Polymerization within a Self‑supported Porous Garnet Framework, *Nano-Micro Lett.* **2024**, *16*, No. 83. <https://doi.org/10.1007/s40820-023-01294-0>.

[S14] Z. Li, S. Zhu, S. Gao, Y. He, H. Ding, D. Yang, H. Yang, P.F. Cao, Fireproof Solid Polymer Electrolyte with Chemically Bonded Phosphorus Toward Stable and Safe Lithium-metal Battery, *Adv. Funct. Mater.* **2024**, *34*, No. 2409836. <https://doi.org/10.1002/adfm.202409836>.

[S15] P. Li, S. Wang, J. Hao, X. Wang, S.M. Hao, Y. Lu, H. Li, W. Zhou, Y. Li, Efficiencies of Various In Situ Polymerizations of Liquid Electrolytes and the Practical Implications for Quasi Solid-state Batteries, *Angew. Chem. Int. Ed.* **2023**, *62*, No. e202309613. <https://doi.org/10.1002/anie.202309613>.

[S16] S. Gao, Y. Pan, B. Li, M.A. Rahman, M. Tian, H. Yang, P.F. Cao, Ultra-stretchable, Ionic Conducting, Pressure-sensitive Adhesive with Dual Role for Stable Li-metal Batteries, *Adv. Funct. Mater.* **2023**, *33*, No. 2210543. <https://doi.org/10.1002/adfm.202210543>.

[S17] Z. Shen, J. Zhong, S. Jiang, W. Xie, S. Zhan, K. Lin, L. Zeng, H. Hu, G. Lin, Y. Lin, S. Sun, Z. Shi, Polyacrylonitrile Porous Membrane-based Gel Polymer Electrolyte by In Situ Free-radical Polymerization for Stable Li Metal Batteries, *ACS Appl. Mater. Interfaces* **2022**, *14*, 41022-41036. <https://doi.org/10.1021/acsami.2c11397>.
